# Beyond traditional methods: Innovative integration of LISS IV and Sentinel 2A imagery for unparalleled insight into Himalayan ibex habitat suitability

**DOI:** 10.1371/journal.pone.0306917

**Published:** 2024-10-21

**Authors:** Ritam Dutta, Lalit Kumar Sharma, Bheem Dutt Joshi, Vineet Kumar, Amira Sharief, Saurav Bhattacharjee, Mukesh Thakur, Dhriti Banerjee, Rajappa Babu

**Affiliations:** 1 Zooological Survey of India, Prani Vigyan Bhawan, Kolkata, West Bengal, India; 2 University of Madras, Chennai, Tamil Nadu, India; 3 Southern Regional Centre, Zoological Survey of India, Chennai, Tamil Nadu, India; 4 WSL, Swiss Federal Research Institute, Zurcherstrasse, Switzerland; Amity University, INDIA

## Abstract

The utilization of satellite images in conservation research is becoming more prevalent due to advancements in remote sensing technologies. To achieve accurate classification of wildlife habitats, it is important to consider the different capabilities of spectral and spatial resolution. Our study aimed to develop a method for accurately classifying habitat types of the Himalayan ibex (*Capra sibirica*) using satellite data. We used LISS IV and Sentinel 2A data to address both spectral and spatial issues. Furthermore, we integrated the LISS IV data with the Sentinel 2A data, considering their individual geometric information. The Random Forest approach outperformed other algorithms in supervised classification techniques. The integrated image had the highest level of accuracy, with an overall accuracy of 86.17% and a Kappa coefficient of 0.84. Furthermore, to delineate the suitable habitat for the Himalayan ibex, we employed ensemble modelling techniques that incorporated Land Cover Land Use data from LISS IV, Sentinel 2A, and Integrated image, separately. Additionally, we incorporated other predictors including topographical features, soil and water radiometric indices. The integrated image demonstrated superior accuracy in predicting the suitable habitat for the species. The identification of suitable habitats was found to be contingent upon the consideration of two key factors: the Soil Adjusted Vegetation Index and elevation. The study findings are important for advancing conservation measures. Using accurate classification methods helps identify important landscape components. This study offers a novel and important approach to conservation planning by accurately categorising Land Cover Land Use and identifying critical habitats for the species.

## Introduction

Satellite imagery is filled with intricate details and plays a substantial role in the distribution of geographical information [1]. The application of satellite and remote sensing imagery offers a range of quantitative and qualitative data, facilitating the efficiency of fieldwork and shortening the research time [2]. The significance of effective analysis and processing of remote sensing images has been on the rise in response to the exponential expansion of remote sensing data [3]. Remote sensing image classification has increasing garnered attention in both research and development because of its wide-ranging potential applications in several domains, including geography, ecology, city planning, forest monitoring, and the military [4]. Nowadays, there has been an increase in the availability of many forms of multispectral data. Multispectral remote sensing data stands out because it combines narrow spectral bands with a comparatively wider bandwidth. This makes it easier to look at the spatial properties of ground substances [5]. Nonetheless, there are constraints associated with depending solely on a single source of satellite data for the precise extraction of ground objects. These limits are a result of similarities in spectral characteristics between various objects or the close proximity of objects in space. Therefore, in order to improve the accuracy of data evaluation, it is necessary to correctly analyse the properties of objects, including their configuration and spatial interconnections, along with their spectral response [5]. In recent years, image fusion has gained increasing importance in image-processing applications due to the wide variety of available acquisition methods [6]. Comprehension of digital image fusion techniques facilitates the interpretation of multiresolution and multi-sensor data, leading to the production of enhanced images that are suited for both human perception and seamless computer analysis tasks, including feature extraction, segmentation, and object recognition [7,8]. This approach has proven advantageous in enhancing the quality of low-resolution data and providing additional information from the same geographical location, complementing data obtained from a single sensor [6]. The domain of image fusion technology has been broadened by the integration of spatial and spectral attributes of remote-sensing images [9]. Nowadays, a notable increase in the interest regarding the utilisation of multi-sensor data fusion for land cover land use (hereafter, LCLU) classification. This surge is mostly driven by the potential to enhance the accuracy of land cover classifications through the assimilation of data from diverse remote sensing sensors that possess varying resolutions [9]. The increasing need for enhanced accuracy in image and data analysis has spurred investigations into the utilisation of multiresolution and multisensor data, along with the development of enhanced techniques for accessing remote sensing data with higher resolutions [10]. Several studies have utilized various data sources, such as radar aperture, hyperspectral and multispectral, to develop LCLU maps [11–18]. The classification of LCLU types frequently entails the application of machine learning algorithms [19–21]. This approach offers significant contributions in evaluating the dynamics of land use, identifying ecosystem services, comprehending the impacts of global climate change, and developing effective land use policies [22–27]. Furthermore, the aim of the study was to assess the performance of different satellite images, including the integrated LISS IV and Sentinel 2A images, in a pilot landscape located in the Jispa valley of the Lahaul and Spiti district, Himachal Pradesh within the Trans-Himalayan region that supports a good population of Himalayan ibex. Our analysis involved comparing the LCLU classifications attained from these abovementioned images. Furthermore, we used the combination of LISS IV’s high spatial resolution and Sentinel 2A’s high spectral resolution to achieve a precise classification of land uses. The mountainous terrain has experienced multifaceted challenges in recent times, particularly in terms of land alteration. These changes have had an impact on the local wildlife, including the Himalayan ibex, which is a "Near Threatened" mammalian species [28]. This species is one of the largest in the genus *Capra* and a member of the Bovidae family [29]. It is native to the Southern Palearctic zone and can be found in a variety of habitats, including as high elevation regions with rocky outcrops, steep slopes, and rough terrain, as well as cold deserts and the foothills of Southern and Central Asia [30]. However, it is mostly limited to mountainous regions of India, such as Himachal Pradesh, Ladakh, and Jammu & Kashmir [29,31,32]. Himalayan ibex have a crepuscular activity pattern, however, this rhythm can be altered by changes in temperature [29,33]. The Asiatic ibex is a sociable species that usually assembles in herds of 6 to 40 animals [29,34–36]. Males and females ibex are sexually dimorphic as well as the body size and horn size and shape also differ in sexes [29]. During the summer, Himalayan ibex migrate to higher elevations, whereas in the winter they travel to lower plains due to the snow covering and limiting their access to food sources [37]. Furthermore, habitat destruction, rapid urbanization, poaching and hunting pose alarming threats to Himalayan ibex [38–40]. Additionally, it has been categorised as a Schedule I species under the Indian Wildlife (Protection) Act, 1972. As a result, the preservation of the habitat of the Himalayan ibex has become a priority in conservation efforts. It is evident that, human-centric development has an impact on the existence of this species in this study landscape. Accordingly, our another objective is to identify the potential suitable habitat for the Himalayan ibex with the help of the classified images and compare the image-derived habitat suitability models. Therefore, we have used Species Distribution Models (hereafter, SDM), which have become a valuable conservation tool, enabling conservation managers to prioritize areas based on species presence and their environmental associations. SDMs help formulate conservation policies, evaluate species richness, estimate the extent of invasion, and predict the probable habitat of a species [41]. SDMs are highly relevant in quantitative ecology [41], which is based on Hutchinson’s ecological niche theory [42]. SDMs do this by carefully looking at how a species interacts with biotic and abiotic factors in a certain area. These models consider factors such as dietary resources, vegetation types, elevation profiles, and climatic elements that influence species interactions within their ecological context [43,44]. The ensemble modeling, uses multiple SDM models instead of a single modelling technique, enhances the accuracy of predicting a species’ geographic range [45–47]. Due to the ambiguity in choosing one strategy from numerous alternatives, ensemble modeling proves to be more effective [48–51]. Subsequently, we utilized the LCLU information from the classified images to model the suitable habitat of the Himalayan ibex in the study area. This study represents the first of its kind to employ SDM at a fine scale, showcasing the potential of image fusion techniques in conservation planning for threatened species. By integrating remote sensing data and SDM, we aimed to gain valuable insights for effective conservation strategies and habitat management of the Himalayan ibex in this region.

## Materials and methods

### Ethical statement

The research was conducted after obtaining research authorization from the Principal Chief Conservator of Forest and Chief Wildlife Warden, Government of Himachal Pradesh, vide letter no. WL/Research Study/WLM/2291 dated 23/07/2018. While the current study does not involve any animal handling, we have exclusively utilised cameras trap data, direct sightings, indirect evidences those are obtained in a non-invasive manner. Therefore, the present study does not necessitate ethical approval.

### Study area

**Fig 1:**
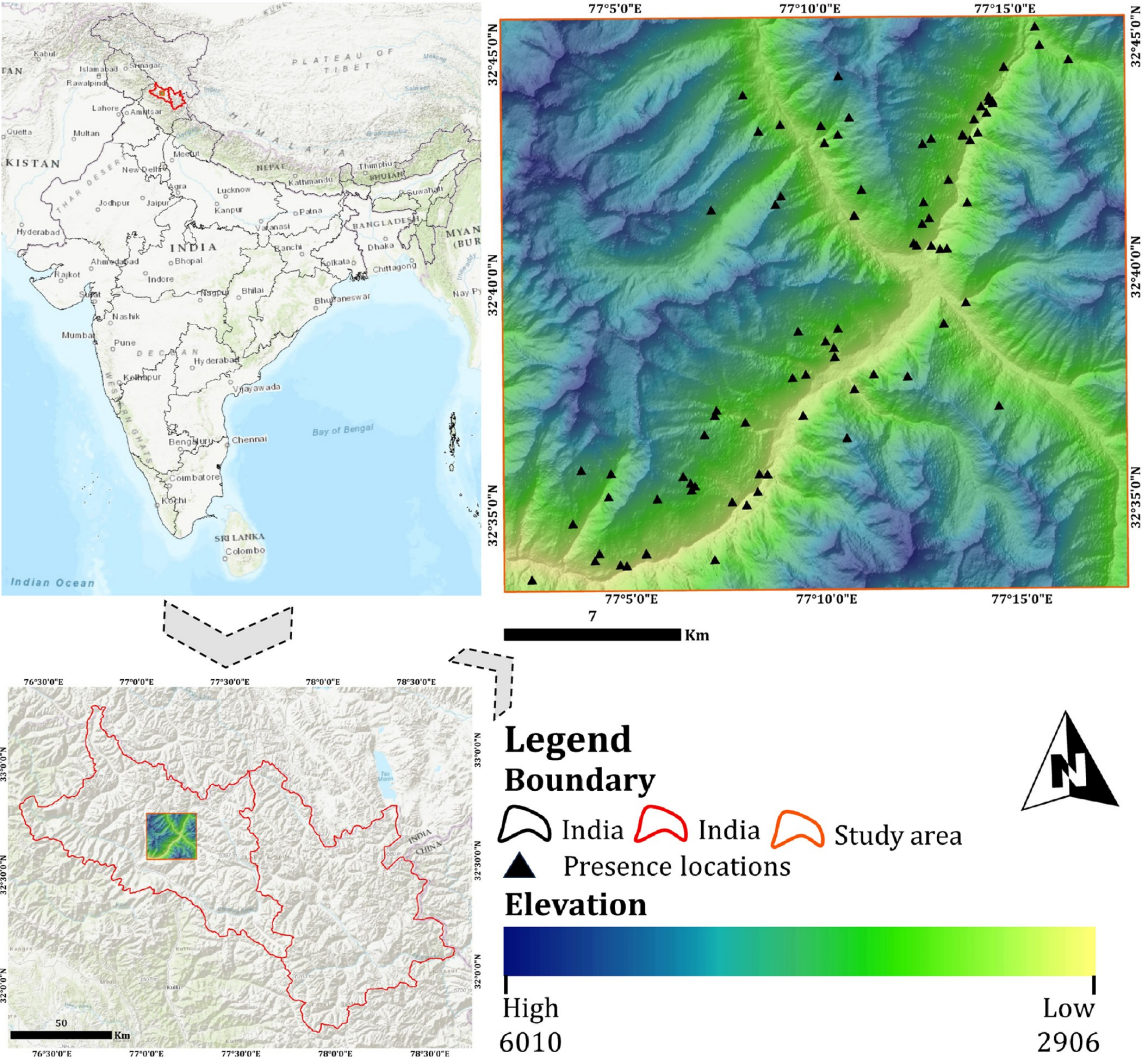
Position of the present study area map. Map showing the position of the study area in India on the upper left and the position of the study area in the Lahaul and Spiti district on the lower left. The presence locations of the Himalayan ibex with the elevation profile of the study area (Jispa Valley, Lahaul, Himachal Pradesh) are shown in the upper right.

The study was carried out in the Jispa valley, which is located in the eastern region of the Lahaul valley within the administrative district of Lahaul and Spiti in the state of Himachal Pradesh, India (Fig 1). The study region spans a total area of 559 km^2^ and falls under the Trans Himalaya Ladakh Mountains (1A) of the Indian biogeographic zones. This region is characterised by its unique geomorphology, which includes high mountains, inclining slopes, and limited vegetation. The major forest types include coniferous forests, alpine and subalpine vegetation, and grassland [52]. The mammalian fauna in the area includes the Himalayan Wolf (*Canis lupus*), Snow leopard (*Panthera uncia*), Red Fox (*Vulpes vulpes*), Himalayan Marmot (*Marmota himalayana*), Himalayan ibex (*Capra sibirica*), mountain weasel (*Mustela altaica*), Pika (*Ochotona roylei*), Himalayan thar (*Hemitragus jamlahicus*), and others [53]. The Lahaul valley is primarily inhabited by the *Lahaulas*, ethnic groups in Lahaul valley [54]. The majority of the population in the study area follow Buddhism and a significant portion of the population belongs to the Schedule Tribe. The region exhibits distinct climatic patterns characterised by two major seasons: dry summers and intense winter with snowfall. Farming serves as a primary source of income, with commercial crops like potatoes, peas, cauliflower, and cabbage being cultivated only in the summer. The presence of the river Bhaga intersects this region, and it holds significant importance due to its support for various cash crops, biodiversity, pasture, and its aesthetic natural environment.

### Data acquisition and processing of these images

In this study, data from two separate satellite sources, namely Linear Imaging Self-Scanning Sensor (LISS) IV and Sentinel 2A, were employed. The LISS IV is a multi-spectral sensor that captures data in three distinct spectral bands with high resolution, *viz*. B2 (ranging from 0.52 μm to 0.59 μm), B3 (ranging from 0.62 μm to 0.68 μm), and B4 (ranging from 0.77 μm to 0.86 μm) with spatial resolution of 5 metres. On the contrary, Sentinel 2A is an optical multispectral imaging mission characterised by its extensive coverage and superior level of resolution. The multispectral optical equipment is equipped with 13 spectral bands, including Visible, Near-Infra-Red, and short-wave Infra-Red. Different spectral bands have different spatial resolutions (10 metres, 20 metres, and 60 metres). The LISS IV imagery was obtained through the Bhoonidhi portal, whereas the Sentinel 2A Level-1C multispectral sensor scenes were acquired from the United States Geological Survey (https://earthexplorer.usgs.gov/) (Table 1).

**Table 1:** Data type and acquisition details for the two satellite imagery.

Satellite/Sensor	Product type	Sensing orbit number	Row	Path	Date of acquisition	Resolution (meter)	Tile No / Product ID (LISS IV)
Sentinel 2A	L1C	105	----	----	2021-10-06	10, 20, 60	T43SFS
Sentinel 2A	L1C	105	----	----	2021-10-06	10, 20, 60	T43SGS
LISS IV	----	----	048	095	2020-09-02	5	221881311

Ensuring better image classification necessitates the meticulous geometric correction and registration of the two images [55]. The Sentinel 2A data underwent pre-processing using the Sentinel Application Platform (SNAP Desktop, Version 6.0.0) in order to improve the resolution of the bands. This was achieved by utilising the highest resolution among the other bands [56]. The bands of the satellite image were stacked using the layer stacked method within the ArcGIS 10.6 environment. The process of image fusion encompasses two primary procedures: (a) the geometric registration of the geometry of two datasets on an image-to-image basis, and (b) the amalgamation of spectral and spatial data to generate a novel dataset that is enhanced and distinct from the original datasets [57]. Image-to-image geometric registration refers to the procedure of aligning and overlaying images that depict the identical scene but were recorded at different times, angles, or using diverse sensors [58]. The satellite images underwent the use of the image-to-image geometric registration approach in order to execute a precise fusion procedure. The approach involved utilising the QGIS Coregistration plugin [59], with the LISS IV image serving as the reference image and the Sentinel 2A image as the target image.

### Integration of the two different satellite images

The nearest neighbour resampling method [60] was utilised in order to mitigate the loss of spectral information in the Sentinel 2A images obtained from SNAP. This approach aimed to attain a spatial resolution of 5 metres, which is comparable to that of the LISS IV images. We utilized ten bands with resolutions of 10 meters and 20 meters for Sentinel 2A. Consequently, we selected the 10 bands with the exception of B1, B9, and B10 from the available 13 bands of Sentinel 2A bands and composited them for the purpose of Sentinel 2A image classification. In contrast, the LISS IV utilized all three available bands. However, for the integration of the image, we combined the three spectral bands of LISS IV with the seven bands of Sentinel 2A. The Sentinel 2A bands were utilised for integration was B2, B5, B6, B7, B8A, B11 and B12. Given that LISS IV consists of bands B2, B3, and B4, representing the Green, Red, and Near-Infrared wavelengths respectively, and Sentinel 2A possesses bands B3, B4, and B8 with comparable spectral resolutions, so the substitution was feasible. Consequently, the integrated image contains a total of ten bands, thereby offering improved spatial and spectral resolution, as well as an augmented band count.

### Image classification

The extraction of relevant information from satellite images necessitate the implementation of a robust and streamlined statistical methodology. The process of image classification entails the assignment of appropriate labels to distinct land cover themes by categorising each pixel in an image or raw data acquired from remote sensing satellites [61,62]. The present study involved the classification of images (namely, LISS IV, Sentinel 2A, and the integrated image) through the application of five supervised machine learning classification techniques: Maximum Likelihood (ML), K-nearest neighbour (KNN), Support Vector Machine (SVM), Gaussian mixed model (GMM), and Random Forest (RF) method. The images were classified into nine distinct classes representing different LCLU types. These classes include agriculture land, sparse vegetation, barren ground, scrubland, juniper patch, settlements, permafrost, water bodies, and roadways. A concise overview of the classes is provided below:

Agricultural land includes cash crops such as peas, potatoes, cabbage, and cauliflower, which are cultivated by villagers. The sparse vegetation includes *Poa* sp., *Rumex* sp., *Primula* sp., and other shrub species. Barren ground characterised by a scarcity of vegetation, a substrate primarily consisting of rocks, and a lack of fertile soil. Scrubland composed of woody plants, which includes species from wild *Rosaceae* family, which characterised by the presence of thorns. The composition of juniper species (such as *Juniperus macropoda*, *Juniperus communis)*, which are typically found growing in close proximity to one another, forming clusters, designated as juniper patch. Consisting of villages, house made of concrete or sometime mud and other human infrastructure, designated as settlements. High elevated areas, characterised by the accumulation of snow maximum period of the year, designated as permafrost. The Bhaga River serves as the primary water source for this landscape, which also intersects the valley. Waterfalls were included in the water body class. The utilisation of road infrastructure for the purpose of vehicular transportation, predominantly constructed using asphalt materials, considered as roadways.

A comprehensive set of 336 polygons was collected during the field survey and then employed in the analysis of all three images. However, some of the obtained geographical coordinates, which were collected during the field survey, are subsequently utilized to generate polygons inside Google Earth Pro using satellite imagery. The collected training dataset included of eight LCLU classes, with the exception of permafrost. During the field survey, a collection of sample points was obtained for accuracy estimation. For each land cover type, a minimum of 90 samples locations were recorded, however, for permafrost we collected sample points by imposing Google earth pro imagery.

The objective of this study was to assess the efficacy of different satellite images, and to determine the accuracy of mapping quantification by applying remote sensing data to actual ground-truth data. Furthermore, the evaluation of accuracy for each class was conducted using an error matrix, which involved comparing the map information with reference data. Moreover, the quality assessment of the three classified images (LISS IV, Sentinel 2A, and the integrated image) was conducted using different accuracy assessments using various metrics, including overall accuracy (OA), Kappa coefficients (κ), and F-measure [63,64]. To evaluate the improvements in the image classification a comparison was made between the integrated image, LISS IV and Sentinel 2A images. The classification of these three images was conducted using the dzetsaka classification tool, the SCP tool in QGIS, and ArcGIS 10.6 [65–67].

### Habitat suitability modelling for Himalayan ibex

#### Occurrence locations of the Himalayan ibex

During the field study conducted between 2019 and 2022, the occurrence of Himalayan ibex was documented through several methods, including camera traps, trail sampling, direct observation, and questionnaire surveys. We deployed 27 camera traps, surveys 46 trails, and directly observed Himalayan ibex 36 times. Additionally, we recorded 131 indirect sightings, which included pallet groups, horns, hoof marks, and information gathered from local residents and herders. The necessity of representative sample was underscored by the challenging topography, characterised by harsh terrain, steep slopes, high mountains, and unpredictable weather conditions within the study area. A total of 167 presence locations were documented, and spatial uncorrelated locations were selected after performing the spatial autocorrelation at a distance of 400 meters, based on the daily movement patterns of Himalayan ibex [32]. Finally, a subset of 82 locations were chosen for the final analysis.

#### Variables preparation and selection

In the context of SDM, the meticulous selection of variables played a pivotal role in ensuring their relevance to the existence of the Himalayan ibex. The Digital Elevation Model (DEM) data obtained from Alos Palsar, at a spatial resolution of 12.5 metres, this was also employed to calculate the slope and aspect. LCLU classes were derived from the classified images with a high level of accuracy. Furthermore, the calculation of radiometric indices pertaining to soil, vegetation, and water was performed using Sentinel 2A images in the SNAP platform. The data underwent rasterization and resampling processes, ensuring a uniform spatial scale of 5 metres, which performed using the spatial analyst extension tool within the ArcGIS 10.6. Moreover, a total of 19 ecologically relevant variables were initially prepared. However, during the final phase of model construction, only variables that exhibited a Pearson correlation coefficient (r) greater than 0.8 and were not correlated with each other were chosen.

#### Selection of modelling techniques and assessment of model performance

The relationship between environmental factors and species is complex and diverse, indicating that single modelling approach is not universally superior [47]. In this study, we employed a range of modelling algorithms, classifying them into three categories: Classification models, Regression models, and Complex models. Specifically, we utilized Multivariate Adaptive Regression Splines (MARS) and Generalized Linear Model (GLM) from the Regression models, Boosted Regression Trees (BRT) from the Classification models, and Maximum Entropy Model (MaxEnt) and Random Forest (RF) from the Complex models. Each model was subjected to 10-fold cross-validation [68,69]. To develop the modelling workflow, we utilized the SAHM module and the VisTrails pipeline, enabling the models to select the most relevant predictors for optimal performance [70,71]. A continuous value ranging from 0–1, derived from each performing model, which, representing the potential habitat suitability for each pixel in the study area. These estimates use to interpreted the potential suitable habitat. Binary maps were employed to determine the predicted habitat suitability, with the minimal training occurrence as the threshold [68].

Three ensemble distribution modelling of Himalayan ibex were predicted utilising the LCLU data derived from the three different sourced classified maps (*viz*. LISS IV, Sentinel 2A, and integrated images) and various topographic and radiometric variables with similar spatial resolutions. The SDM prediction utilizing different sourced images have their respective spatial scale. Final ensemble maps were generated by averaging the binary outcome (0 or 1) of the participating models. Furthermore, the count surface illustrated the level of agreement between the participatory models, with a score of 0 indicating no agreement and a score of 5 representing total agreement, in relation to suitability estimation [69]. In order to assess the efficacy of SDMs, several metrics were employed, namely the area under the receiver operating characteristic curve (AUC), Cohen’s Kappa, True Skill Statistic (TSS), Proportion Correctly Classified (PCC), sensitivity, and specificity. The metrics were utilised in a thorough manner to evaluate the performance of the five models [72–76]. The AUC proved to be highly advantageous as a threshold-independent metric for the evaluation of models [77–80]. Furthermore, we utilised the minimal training presence threshold to evaluate the specificity and sensitivity metrics, which are reliant on the chosen threshold [81]. The selection of the ensemble model was determined by establishing a criterion that required the AUC value of the cross-validation (CV) dataset to exceed 0.75. In order to evaluate the significance of variables, we computed the average AUC, which quantifies the ratio of AUC values to the total number of model iterations for each individual model [69].

## Result

### Classification of Land class and Land use

**Fig 2:**
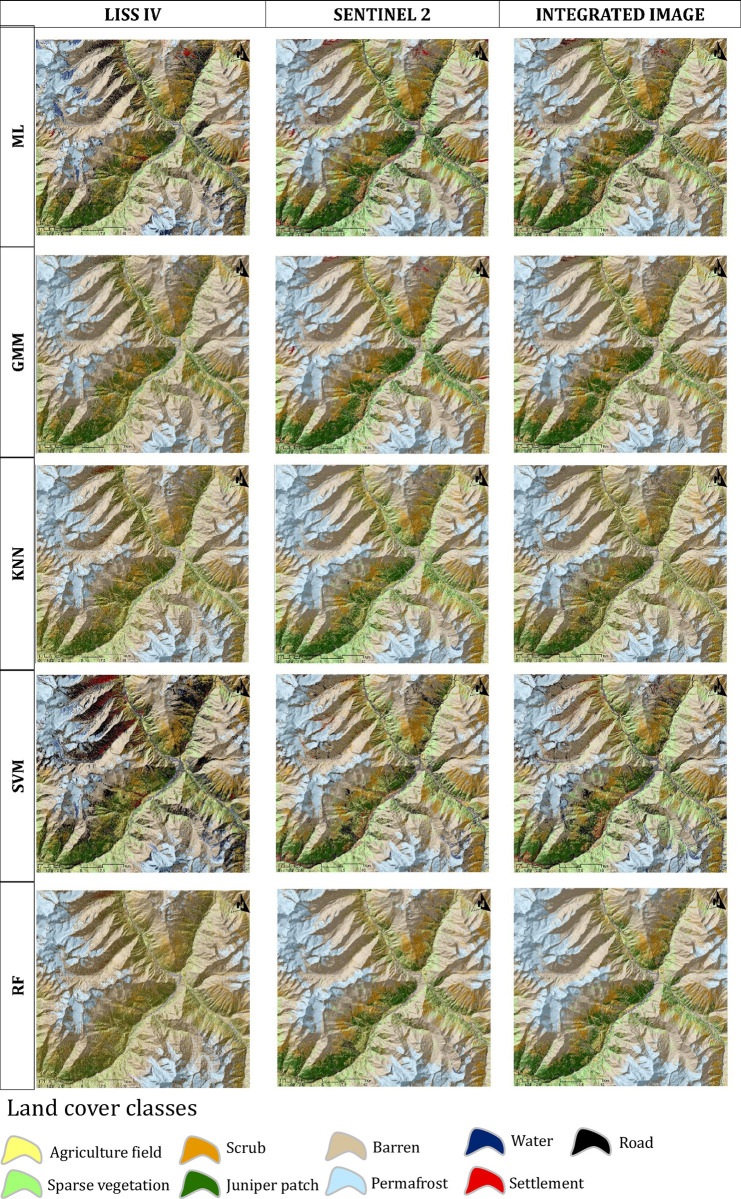
Classified maps from LISS IV, Sentinel 2A and Integrated images using ML (Maximum Likelihood), GMM (Gaussian mixed model), KNN (K-nearest neighbour), SVM (Support Vector Machine) and RF (Random Forest) classifier algorithms.

Using five different supervised classification algorithms for each image, the classification results of the three images came up with nine LCLU classes (Fig 2). Extracting these LCLUs from LISS IV data, with its fine resolution, was challenging as it fails to discriminate different classes simultaneously. However, the integrated image (LISS IV and Sentinel 2A) provided us with an improved classification with better accuracy in the identification of LCLUs features. The classification accuracy was found to be better among the other two different satellite images. The results indicate that the classification accuracy of the LISS IV image was not satisfactory, with no classifying algorithm performing well. The SVM algorithm achieved the highest overall accuracy i.e., 60 and κ statistic of 0.56, while the GMM algorithm performed the poorest with an overall accuracy value of < 50, however, both achieving moderate accuracy as per Landis and Koch 1977 (Table 2 and S1 Table). In addition, the ML, RF, and GMM classifier, the overall accuracy scores were found to be below 60, indicating suboptimal performance. Similarly, the κ statistic values were also observed to be unsatisfactory (Table 2). For Sentinel 2A imagery, the RF model exhibited the highest OA i.e., 80.24 and κ statistic score 0.78, while the KNN algorithm illustrate the lowest performance, showing substantial agreement (Table 2, Fig 2, S1 Table). Other algorithms, namely SVM, ML, and GMM, demonstrated OA scores exceeding 70, along with κ statistic scores beyond 0.7, suggesting a substantial accuracy (Table 2, Fig 2, S1 Table). Interestingly, the integrated image demonstrated higher classification accuracy compared to the individual LISS IV and Sentinel 2A images (Table 2, Fig 2, S1 Table). Furthermore, the integrated image classified by the KNN and RF algorithms achieved OA scores ≥ 80 as well as a κ statistic ≥ 0.80, which was the best among all other classifications (Table 2, Fig 2 and S1 Table). The SVM, ML, and GMM classifiers predicted OA scores of 76.79, 71.72, and 67.77, respectively, and κ statistic scores of 0.74, 0.68, and 0.64, respectively, on the integrated image (Table 2, Fig 2, S1 Table). However, to identify each feature class accuracy, we calculated, F-measure [82], which show the class classification error (Table 3). Furthermore, the best feature classification achieved by the RF classification on the integrated image and the GMM classification achieved a lesser accuracy level on the LISS IV. The classification accuracy of the Barren, Scrub, Settlement, road, and Permafrost classes is the lowest in the GMM classification on the LISS IV than the others, which made this map least accurate map (Table 3).

**Table 2:** Accuracy assessment of the three different sourced classified images. LISS IV, Sentinel 2A and Integrated images classified by five supervised classifiers, namely, ML (Maximum Likelihood), GMM (Gaussian mixed model), KNN (K-nearest neighborhood), SVM (Support Vector machine) and RF (Random Forest). The evaluation metrics are Overall accuracy, Kappa (κ) statistic.

Accuracy Assessment	Image type	ML	GMM	KNN	SVM	RF
Overall accuracy	LISS IV	57.41	48.77	55.31	60.62	56.42
	Sentinel 2A	73.08	71.35	70.24	76.54	80.24
	Integrated	71.73	67.78	81.85	76.79	86.17
Kappa (κ) statistic	LISS IV	0.52	0.42	0.50	0.56	0.51
	Sentinel 2A	0.70	0.68	0.67	0.74	0.78
	Integrated	0.68	0.64	0.80	0.74	0.84

**Table 3:** Class accuracy metrics of three different sourced classified images. Using F-measure LISS-IV, Sentinel 2A and Integrated classified image class accuracy evaluated.

	LCLU Class	LISS-IV	Sentinel 2A	Integrated		LCLU Class	LISS-IV	Sentinel 2A	Integrated
Random forest	Agriculture	53	75	85	Maximum Likelihood	Agriculture	48	67	67
	Sparse vegetation	50	78	84		Sparse vegetation	63	55	49
	Barren	60	76	80		Barren	66	71	72
	Scrub	49	86	80		Scrub	51	68	61
	Juniper patch	50	74	85		Juniper patch	52	73	72
	Settlement	44	78	85		Settlement	28	65	65
	Permafrost	88	99	100		Permafrost	89	100	99
	Water	76	94	98		Water	82	96	95
	Road	16	57	80		Road	29	60	63
	LCLU Class	LISS-IV	Sentinel 2A	Integrated		LCLU Class	LISS-IV	Sentinel 2A	Integrated
Gaussian mixed model	Agriculture	46	66	63	K-nearest neighbour	Agriculture	50	57	74
	Sparse vegetation	36	58	51		Sparse vegetation	59	74	83
	Barren	55	67	64		Barren	58	72	83
	Scrub	44	70	63		Scrub	48	78	76
	Juniper patch	48	72	74		Juniper patch	56	65	76
	Settlement	12	65	57		Settlement	16	43	72
	Permafrost	78	99	98		Permafrost	87	99	100
	Water	69	94	94		Water	78	92	96
	Road	4	41	34		Road	4	31	75
	LCLU Class	LISS-IV	Sentinel 2A	Integrated					
Support Vector Machine	Agriculture	54	51	54					
	Sparse vegetation	62	74	76					
	Barren	72	86	83					
	Scrub	52	78	78					
	Juniper patch	51	74	72					
	Settlement	38	64	63					
	Permafrost	89	98	98					
	Water	83	94	95					
	Road	41	70	73					

#### Habit suitability ensemble model

**Fig 3:**
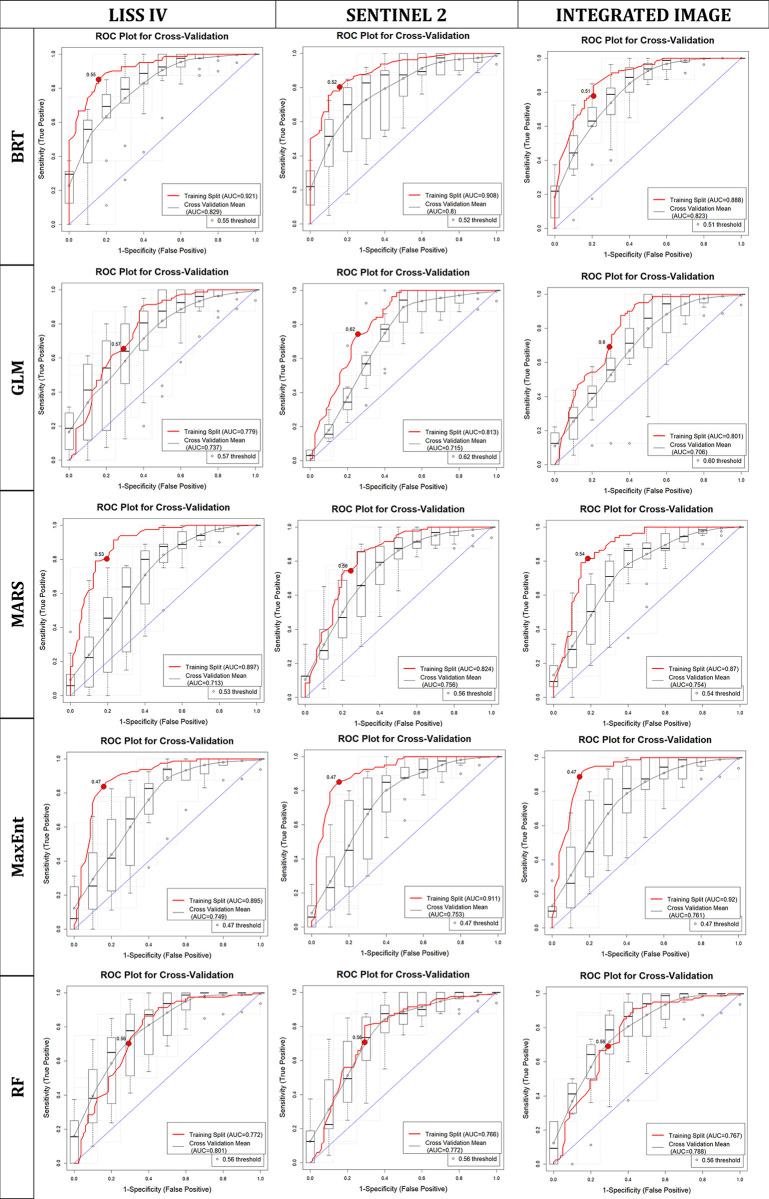
AUC plots. AUC plots of the five algorithms on different sourced images to predict species distribution model.

**Fig 4:**
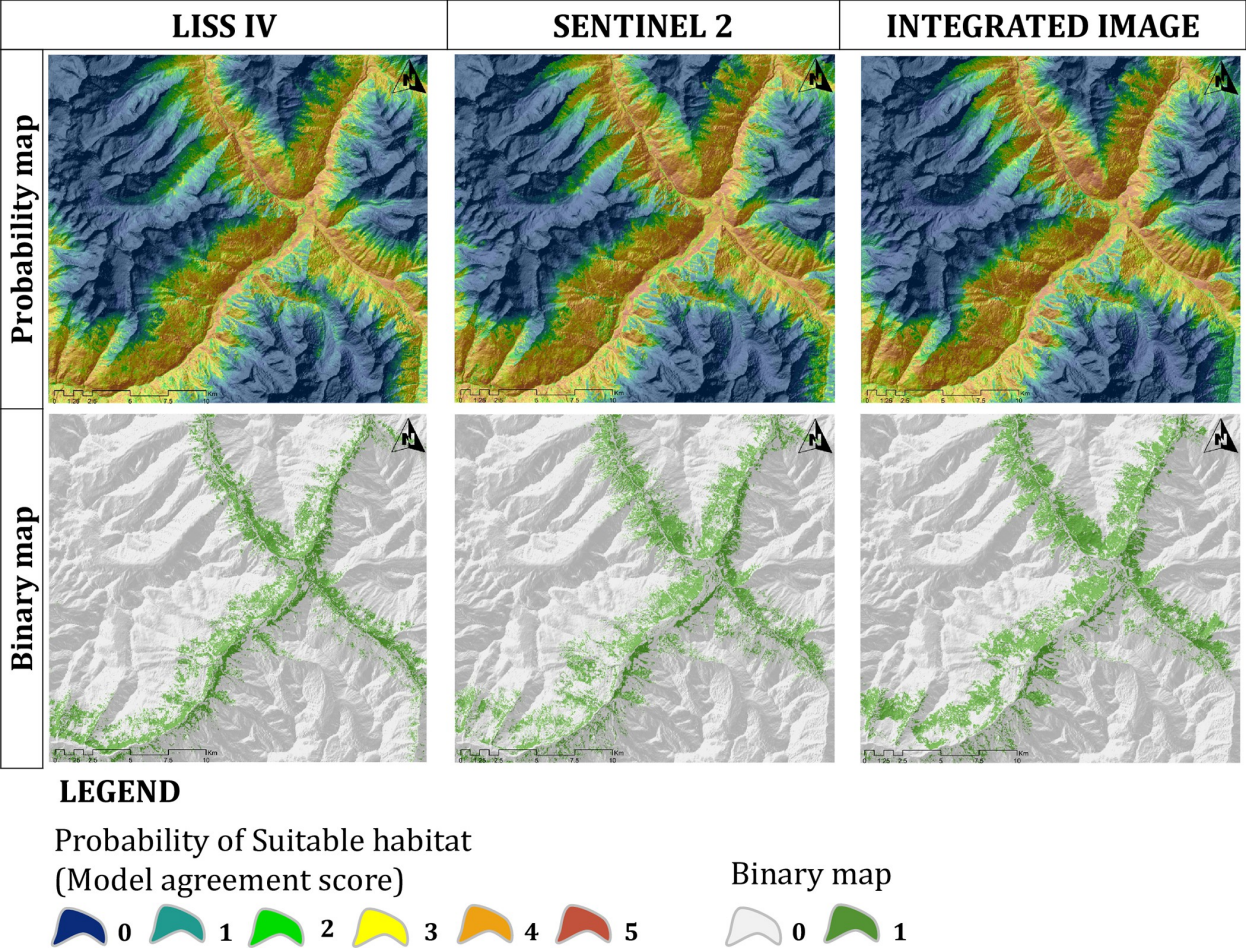
Probability (model agreement maps) maps and binary maps. The maps are derived from three different sourced images generated LCLU to predict species distribution model of Himalayan ibex.

The final SDM model was built separately using uncorrelated variables from three image derived LCLU variables, topographic and radiometric variables (S2 Table) (S1 Fig). The AUC values of five different modelling algorithms ranged from 0.77 to 0.92 when using LCLU classes derived from the LISS IV classified image, from 0.77 to 0.91 when using LCLU classes from the Sentinel 2A classified image, and from 0.77 to 0.92 when using LCLU classes from the integrated classified image for training data sets, indicating excellent model performance (Table 4, Fig 3). Other performance metrics, such as TSS, PCC, Cohen’s Kappa, specificity, and sensitivity, also showed good performance by the models (Table 4) (S2 and S3 Figs). We developed the final ensemble model and ensemble count maps considering each participation model that met the AUC requirement of 0.75 and above (Figs 3 and 4). The model with integrated image derived LCLU combined with other variables resulted in 78.42 km^2^, which is the highest predicted suitable area, followed by 72.42 km^2^ predicted using the Sentinel-2A driven LCLU along with other variables and 34.77 km^2^ is the common area where all the image derived models predict suitability (Table 5, Fig 4, S6 Fig). The performance of all models using different images is similar, as the radiometric (SAVI) variable tops among the all variables in the maximum models (S4 Fig). Furthermore, the SDM using RF classified integrated image derived LCLU, which demonstrated the Juniper patch, elevation, aspect, and settlement as significant factors (S4 Fig). In the same way, the SDM using RF classified Sentinel 2A image derived LCLU, which showed that similar variables were important, like the SDM using integrated image derived LCLU (S4 Fig). However, in the case of SDM, utilizing SVM classified LISS IV image derived LCLU shows the influence of elevation, water, aspect, Juniper patch, and slope variables (S4 Fig). Different LCLU variables exhibited varying degrees of significance in the three types of images, with water class being more significant in the LISS IV image and Juniper patches showing greater significance in the Sentinel 2A and integrated image derived models (S4 Fig). When analysing response curves for these variables, we observed high peaks in areas with prevalent Himalayan ibex occurrences, and the likelihood values decreased as the distance from these areas increased (S5 Fig). This result suggests that these variables perform crucial role in shaping the Himalayan ibex suitable habitats (S5 Fig).

**Table 4:** Evaluation metrics to evaluate the efficiency of the participating distribution models for Himalayan ibex in the study landscape. Participating models are BRT (Boosted Regression Tree), GLM (Generalized Linear Model), MARS (Multivariate adaptive regression splines), MAXENT (Maximum Entropy Model), RF (Random Forest) and the efficiency of the models evaluated by AUC (area under the receiver operator curve), PCC (Proportion Correctly Classified), sensitivity, specificity, Cohen’s kappa and TSS (True Skill Statistic). CV mean (Cross Validation) data used for model evaluation and train split used for the model, which was assessed by model evaluation. Three data type used for SDM are, LISS IV derived LCLU used for this model building, Sentinel 2A derived LCLU used for this model building and Integrated image derived LCLU used for this model building.

Utilising the LISS IV derived LCLU classes						
Model	AUC	PCC	Sensitivity	Specificity	Kappa	TSS
BRT	0.92	84.67	0.85	0.84	0.69	0.69
GLM	0.78	68.1	0.65	0.71	0.36	0.36
MARS	0.9	80.37	0.8	0.8	0.61	0.61
MAXENT	0.9	83.95	0.84	0.84	0.68	0.68
RF	0.77	70.55	0.7	0.71	0.41	0.41
Utilising the Sentinel 2A derived LCLU classes						
Model	AUC	PCC	Sensitivity	Specificity	Kappa	TSS
BRT	0.91	82.32	0.8	0.84	0.65	0.65
GLM	0.81	74.39	0.74	0.74	0.49	0.49
MARS	0.82	75	0.74	0.76	0.5	0.5
MAXENT	0.91	85.28	0.85	0.85	0.71	0.71
RF	0.77	70.73	0.71	0.71	0.41	0.41
Utilising the Integrated image derived LCLU classes						
Model	AUC	PCC	Sensitivity	Specificity	Kappa	TSS
BRT	0.89	78.53	0.78	0.79	0.57	0.57
GLM	0.8	69.94	0.69	0.71	0.4	0.4
MARS	0.87	81.6	0.81	0.82	0.63	0.63
MAXENT	0.92	87.04	0.89	0.85	0.74	0.74
RF	0.77	69.94	0.69	0.71	0.4	0.4

**Table 5:** Area of suitable habitat of Himalayan ibex. Calculation from different classified image (viz. LISS IV, Sentinel 2A and Integrated image) and the predicted suitable common area between the three-classified image. The area calculated from the highest model agreement (units in km^2^).

	LISS-IV	Sentinel 2A	Integrated
LISS-IV	63.80	41.51	44.92
Sentinel 2A	41.51	72.42	54.14
Integrated	44.92	54.14	78.42

## Discussion

The LCLU classification derived from remotely sensed data has been excessively explored in the domain of image analysis. Researchers have utilized various satellite sensors and classification algorithms to extract valuable information from satellite imagery for land cover mapping and monitoring [83,84]. High-resolution sensors like LISS IV and multispectral sensors like Sentinel 2A have been broadly employed for LCLU classification due to their capabilities to capture detailed spectral and spatial information [82,85]. The analysis of land cover classification deal with a major challenge which is the accurate discrimination of different land cover classes, especially in complex landscapes with heterogeneous land cover types [86]. Previous studies have highlighted the limitations of using individual satellite images for LCLU classification due to their varying spatial and spectral resolutions of different sensors [57]. This limitation can lead to misclassification and reduced overall accuracy. To address this challenge, image fusion techniques evolve to integrate images of different sensors which improve the accuracy of land cover classification [57]. Image fusion involves combining spectral and spatial data from multiple images to create a new dataset that benefits from the strengths of each sensor. This aspect holds significance in the context of biodiversity monitoring as it facilitates the differentiation of various ecosystems, vegetation categories and geomorphological structures [87]. In our study, the integration of LISS IV and Sentinel 2A images allowed for improved classification accuracy, as demonstrated by the higher overall accuracy and κ statistic values compared to the individual images. In total, fifteen images were compared with each other by qualitative measures (Tables 2 and 3). Moreover, the choice of classification algorithms also significantly influences the accuracy of LULC mapping [88]. The mentioned classifier algorithms have been widely used in the field of remote sensing applications and have shown varying levels of accuracy [88–90]. The study findings illustrate a significant enhancement in classification accuracy when employing the RF, on the integrated image (Tables 2 and 3) (Fig 2). Previous studies highlighted that RF algorithm can classify images most effectively with great accuracy, as well as with less noise [82,91,92]. The accurate classification of LCLU is vital for many applications, including ecological assessments, urban planning, and management of natural resources [83,87,93]. Accurate information of land cover is essential for monitoring changes in the landscape, understanding habitat fragmentation, and identifying critical habitats for endangered species [94]. Hence, the present results, with improved classification accuracy using integrated images, contribute to the existing knowledge of image analysis and its application in land cover mapping. Furthermore, it is essential to consider some limitations and challenges in land cover classification. Image classification is subject to uncertainties arising from sensor calibration, atmospheric effects, and class confusion due to spectral similarity [83]. Additionally, ground truthing data for accurate model validation and evaluation can be challenging to obtain, especially in remote and inaccessible areas [95]. Addressing these challenges and further refining classification techniques will enhance the accuracy and reliability of LCLU mapping using remotely sensed data. We demonstrated the usefulness of integration image in improving the accuracy of the LCLU. The integration of LISS IV and Sentinel 2A images, coupled with a range of classification algorithms, results in more accurate land cover maps. These findings align with existing research on the benefits of image integration in remote sensing applications. The improved land cover information obtained through this approach has significant implications for ecological monitoring, conservation planning, and sustainable land use management.

The classification provides valuable data on the distribution of different LCLU types, which is essential for understanding the habitat preferences of the wild species and, in the present case the Himalayan ibex. This information is a fundamental component of the SDM, as it allows for the identification of the different land cover types that the species prefers or avoids. The habitat suitability model for the Himalayan ibex, obtained through the utilisation of the most effective classification algorithm on three images, indicates a discernible alteration in the suitable habitat (Table 5, Fig 4 and S6 Fig). Based on the findings of this study, it can be inferred that within this challenging topographical environment, the species exhibit a multifaceted relationship with their habitat. The species preference within the environment is not solely determined by land classes, but is also significantly influenced by the physical characteristics of the terrain (S4 and S5 Figs). SAVI, Juniper Patch, Elevation, Aspect, water, and settlement are the common variables that have the most influence in the prediction of suitable habitat utilizing three different sourced images (S4 Fig). In addition to these variables, the sparse vegetation and barren LCLU classes have a significant impact when the model is run using the LCLU obtained from the integrated image, which is the most accurately classified image (S4 Fig). However, studies have revealed that the most important characteristic for the habitat of Himalayan ibex is terrain geomorphometry, which includes factors like as slope, elevation, and ruggedness [96–99]. This finding illustrates that in this study landscape, the topographical features of the terrain play a crucial role, with the SAVI (Soil Adjusted Vegetation Index) which exerting a significant influence on the prediction of the habitat for Himalayan ibex. However, the SDM evaluation matrices show similar results for all three image derived images. One possible explanation for this could be a thorough examination of the variables’ relative importance. The most important variables are SAVI, elevation, water, aspect, juniper patch, and slope in the SDM using the poorly classified LISS IV derived LCLU and other variables (S4 Fig). The result illustrates the topographical variables play a crucial role to build the SDM while using the LISS IV classified LCLU. Therefore, the poorly classified image also gives similar predictions like best classified images. SAVI is used to adjust the NDVI in areas with sparse vegetation to account for the influence of soil reflectance. The LISS IV image, which has been misclassified, depicts the region with the least suitability, followed by the classified image from Sentinel 2A (Table 5) (Fig 4 and S6 Fig). Himalayan ibex habitat selection is significantly influenced by elevation and slope with barren areas, as these factors provide escape routes from predators [96], these variables also play important role in our suitable habitat prediction model for Himalayan ibex in this present study area (S4 Fig). The predicted suitable area of the Himalayan ibex mainly governed by topographical features and the vegetation [97,98]. Furthermore, the vegetation is distributed differently in different aspect and slope patterns in this rugged terrain, and the selection of the habitat by Himalayan ibex heavily depends on that association [97,98].

However, previous findings on satellite image fusion illustrate that this enhances the species and ecosystem monitoring, as well as it can identify potential risks to biodiversity [100–107]. The best-classified image proves to be particularly useful in the SDM for several reasons. Firstly, a well-classified image provides precise and reliable information on the distribution of different LCLU types, and secondly, this accurate LCLU mapping is crucial for creating an effective model that can distinguish suitable and unsuitable habitat areas for the Himalayan ibex. The improved precision of the best-classified image ensures that the resulting SDM predictions are more reliable and can better inform conservation efforts.

## Conclusion

In this study, we conducted an extensive comparative analysis of three distinct satellite images, *viz*. LISS IV, Sentinel 2A, and an integrated image, using five classification algorithms to enhance LCLU analyses and predict the Himalayan ibex suitable habitat. Our primary aim was to determine the most effective image and classification approach for accurate habitat mapping in the challenging and diverse landscape of the study area. The complexity of the varied environment can often make it challenging to differentiate between classes. However, the utilisation of the RF method on the integrated image greatly improved the accuracy of class mapping. The F-measure reveals that the classification of LCLU classes in LISS IV and Sentinel 2A images exhibits poor accuracy scores, furthermore, the implementation of integration techniques has been shown to enhance their accuracy. In a nutshell, the integrated image presented higher levels of accuracy compare to other two satellite imagery. In terms of both visual and qualitative examination of those classified images, RF exhibited the most accurate outcomes on integrated image, while the GMM classification performed the least effectively on the LISS IV image. However, it is apparent that the Sentinel 2A image has satisfactory performance in classifying ground objects. On the other hand, the LISS IV image could not be effectively classified by any classifier method.

Moreover, this study provides additional support for the effectiveness of ensemble SDM modelling in the prioritisation of conservation methods and management. It is advisable to employ an ensemble model rather than relying solely on a single modelling technique, especially when dealing with species that inhabit intricate habitats. The incorporation of topographic and radiometric variables in addition to LCLU data proved indispensable for accurately predicting the species’ suitable habitat, considering the diverse geomorphological characteristics of the study area. The Himalayan ibex holds great importance as a crucial ecosystem modifier, as exemplified by its function as a primary food source of the snow leopard, the apex predator in the Trans Himalayan region [108,109]. Furthermore, like other browsers, this species can remove vegetation by trampling which creates wide spaces that help in germination with less competition, additionally, their pallets act as natural fertilizer, enriching the soil with nutrients for seedlings [110,111]. Large herbivores (body mass more than or equals to 100 kilogram) considered as ecological engineers and keystone species due to their enormous role in ecological processes and these processes arouse by their size, behaviour and abundance [112]. They have crucial roles in shaping landscapes, as they have nourished and maintained ecologically diverse and productive ecosystems for thousands of years [113,114]. Furthermore, the cultural value of the Himalayan ibex is deeply rooted in the beliefs and traditions of the indigenous communities residing in the present study landscape. Hence, it can be inferred from the present study that the integration of two satellite images can accurately classifying LCLU, thereby facilitating SDM for the assessment of a species’ suitable habitat. The result from such integrated images can be of great use in conservation planning and management of ecosystem with reference to species specific habitat for the long-term viability of wildlife species.

## Supporting information

S1 Table Error matrix of the accuracy assessment for Land Cover Land Class (LCLU) of the three different sourced classified images by five different classification algorithms. (PDF)

S2 Table Independent variables utilised to forecast Himalayan ibex ensemble distribution in Jispa valley landscape using three different sourced images. (PDF)

S1 Fig Correlation matrix among the all variables and final variable selection based on <0.8 for the predicting final species distribution models of Himalayan ibex. (PDF)

S2 Fig Different models confusion matrix: Represents the confusion matrix for each of the five models. The observed vs. predicted results are presented.
(PDF)

S3 Fig Calibration plots and residual plots of the five distribution models. (PDF)

S4 Fig Variable significance: Each predictor is permuted for training and cross-validation data, the change in AUC is used to assess the variable’s significance, when using the LCLU derived from (a) LISS IV classified image, (b) Sentinel 2A classified image, (c) Integrated image classified image along with other topographic and radiometric variables. (PDF)

S5 Fig Representation of the response curves of the selected variables used by different SDMs. (PDF)

S6 Fig Variance in suitable habitat prediction of Himalayan ibex by three different sourced images generated LCLU along with topographic and radiometric variables. (TIF)

S1 File (CSV)
