# Supplementary material for: Beyond traditional methods: Innovative integration of LISS IV and Sentinel 2A imagery for unparalleled insight into Himalayan ibex habitat suitability
Source: PLoS One. 2024 Oct 21;19(10):e0306917. doi: 10.1371/journal.pone.0306917 (PMC11493286; doi:10.1371/journal.pone.0306917)
Supplement: S1 Table — (PDF) [file pone.0306917.s001.pdf]

S1 Table: Error matrix of the accuracy assessment for Land Cover Land Class (LCLU) of the three different sourced classified images by five different classification algorithms. LISS IV, Sentinel 2A and Integrated images classified by five supervised classifiers, namely, ML (Maximum Likelihood), GMM (Gaussian mixed model), KNN (K-nearest neighborhood), SVM (Support Vector machine) and RF (Random Forest). Error matrix of LISS IV image classification using (A) ML, (B) GMM, (C) KNN, (D) SVM, (E) RF classifier model. Error matrix of Sentinel 2A image classification using (F) ML, (G) GMM, (H) KNN, (I) SVM, (J) RF classifier model. Error matrix of Integrated image classification using (K) ML, (L) GMM, (M) KNN, (N) SVM, (O) RF classifier model.

(A)

Classification of the LISS IV image using ML

		**Reference classes**									
		Agriculture	Sparse vegetation	Barren	Scrub	Juniper patch	Settlement	Permafrost	Water	Road	Total
**Classified classes**	Agriculture	45	4	0	9	3	28	0	1	6	96
	Sparse vegetation	2	46	0	2	3	2	0	0	1	56
	Barren	0	7	77	4	3	8	6	7	32	144
	Scrub	18	10	4	49	10	7	0	0	4	102
	Juniper patch	16	19	1	20	59	7	0	1	15	138
	Settlement	8	3	3	2	0	18	0	1	3	38
	Permafrost	0	0	1	0	0	0	75	2	0	78
	Water	0	0	0	0	0	1	9	73	6	89
	Road	1	1	4	4	12	19	0	5	23	69
	Total	90	90	90	90	90	90	90	90	90	810

Overall accuracy (OA): 57.41; Kappa coefficients (κ): 0.52; SE of kappa: 0.02; Weighted Kappa= 0.51

95% confidence interval: From 0.48 to 0.56

Conclusion: The level of agreement is considered as 'Moderate'.

(B)

Classification of the LISS IV image using GMM

**Classified classes**		**Reference classes**									
		Agriculture	Sparse vegetation	Barren	Scrub	Juniper patch	Settlement	Permafrost	Water	Road	Total
	Agriculture	40	3	0	1	2	34	0	1	2	83
	Sparse vegetation	1	21	1	0	3	0	0	0	0	26
	Barren	1	10	82	15	9	20	6	12	51	206
	Scrub	29	23	2	50	14	11	0	0	7	136
	Juniper patch	16	30	0	22	62	15	0	3	21	169
	Settlement	3	1	0	1	0	6	0	1	0	12
	Permafrost	0	2	5	1	0	2	84	25	6	125
	Water	0	0	0	0	0	1	0	48	1	50
	Road	0	0	0	0	0	1	0	0	2	3
	Total	90	90	90	90	90	90	90	90	90	810

Overall accuracy (OA): 57.41; Kappa coefficients (κ): 0.42; SE of kappa: 0.02; Weighted Kappa= 0.41

95% confidence interval: From 0.39 to 0.46

Conclusion: The level of agreement is considered as 'Moderate'.

(C)

Classification of the LISS IV image using KNN

Classified classes	Agriculture	Sparse vegetation	Barren	Scrub	Juniper patch	Settlement	Permafrost	Water	Road	Total
	**Reference classes**									
Agriculture	46	1	0	4	3	35	0	2	2	93
Sparse vegetation	3	43	0	2	4	4	0	0	1	57
Barren	1	14	82	10	6	16	3	12	48	192
Scrub	27	18	3	55	10	17	0	0	8	138
Juniper patch	13	13	0	19	67	9	0	2	25	148
Settlement	0	0	0	0	0	8	0	1	2	11
Permafrost	0	1	5	0	0	1	87	15	1	110
Water	0	0	0	0	0	0	0	58	1	59
Road	0	0	0	0	0	0	0	0	2	2
Total	90	90	90	90	90	90	90	90	90	810

Overall accuracy (OA): 55.31; Kappa coefficients (κ): 0.50; SE of kappa: 0.02; Weighted Kappa= 0.46

95% confidence interval: From 0.46 to 0.53

Conclusion: The level of agreement is considered as 'Moderate'.

(D)

Classified classes	Agriculture	Sparse vegetation	Barren	Scrub	Juniper patch	Settlement	Permafrost	Water	Road	Total
	**Classification of the LISS IV image using SVM**									
	**Reference classes**									
Agriculture	58	6	0	15	5	33	0	1	5	123
Sparse vegetation	3	48	2	2	6	4	0	0	0	65
Barren	0	6	72	3	1	2	8	3	14	109
Scrub	13	13	2	48	7	6	0	0	4	93
Juniper patch	13	12	1	18	55	6	0	3	16	124
Settlement	3	2	0	0	7	27	1	1	11	52
Permafrost	0	0	0	0	0	0	74	3	0	77
Water	0	0	1	0	0	1	7	74	5	88
Road	0	3	12	4	9	11	0	5	35	79
Total	90	90	90	90	90	90	90	90	90	810

Overall accuracy (OA): 60.62; Kappa coefficients (κ): 0.56; SE of kappa: 0.02; Weighted Kappa= 0.57

95% confidence interval: From 0.52 to 0.60

Conclusion: The level of agreement is considered as 'Moderate'.

(E)

Classification of the LISS IV image using RF

Classified classes	Reference classes: Agriculture	Sparse vegetation	Barren	Scrub	Juniper patch	Settlement	Permafrost	Water	Road	Total
Agriculture	48	2	0	7	2	24	0	2	6	91
Sparse vegetation	4	39	2	2	7	4	0	2	5	65
Barren	2	11	78	9	9	11	2	8	42	172
Scrub	20	21	3	52	11	9	0	0	6	122
Juniper patch	13	16	3	19	58	13	0	1	17	140
Settlement	2	0	0	0	1	27	0	0	2	32
Permafrost	0	0	4	0	1	0	88	18	0	111
Water	1	0	0	0	0	1	0	59	4	65
Road	0	1	0	1	1	1	0	0	8	12
Total	90	90	90	90	90	90	90	90	90	810

Overall accuracy (OA): 56.42; Kappa coefficients (κ): 0.51; SE of kappa: 0.02; Weighted Kappa= 0.50

95% confidence interval: From 0.47 to 0.55

Conclusion: The level of agreement is considered as 'Moderate'.

(F)

Classification of the Sentinel 2A image using ML

		Reference classes									
		Agriculture	Sparse vegetation	Barren	Scrub	Juniper patch	Settlement	Permafrost	Water	Road	Total
**Classified classes**	Agriculture	62	11	0	5	4	12	0	0	2	96
	Sparse vegetation	3	42	7	7	3	0	0	0	0	62
	Barren	1	4	72	12	1	3	0	5	15	113
	Scrub	2	19	10	65	2	0	0	0	2	100
	Juniper patch	9	13	0	1	70	1	0	0	8	102
	Settlement	11	0	1	0	0	54	0	0	10	76
	Permafrost	0	0	0	0	0	0	90	0	0	90
	Water	0	0	0	0	0	1	0	84	0	85
	Road	2	1	0	0	10	19	0	1	53	86
	Total	90	90	90	90	90	90	90	90	90	810

Overall accuracy (OA): 73.08; Kappa coefficients (κ): 0.70; SE of kappa: 0.02; Weighted Kappa= 0.71

95% confidence interval: From 0.66 to 0.73

Conclusion: The level of agreement is considered as 'Substantial'

(G)

Classification of the Sentinel 2A image using GMM

Classified classes	Reference classes									
	Agriculture	Sparse vegetation	Barren	Scrub	Juniper patch	Settlement	Permafrost	Water	Road	Total
Agriculture	58	7	0	0	2	16	0	0	4	87
Sparse vegetation	1	40	0	3	4	0	0	0	0	48
Barren	3	6	84	16	2	6	0	7	36	160
Scrub	7	23	5	71	5	0	0	0	2	113
Juniper patch	14	14	0	0	76	2	0	0	14	120
Settlement	7	0	1	0	0	50	0	1	5	64
Permafrost	0	0	0	0	0	0	90	0	1	91
Water	0	0	0	0	0	1	0	81	0	82
Road	0	0	0	0	1	15	0	1	28	45
Total	90	90	90	90	90	90	90	90	90	810

Overall accuracy (OA): 71.35; Kappa coefficients (κ): 0.68; SE of kappa: 0.02; Weighted Kappa= 0.65

95% confidence interval: From 0.64 to 0.71

Conclusion: The level of agreement is considered as 'Substantial'

(H)

Classification of the Sentinel 2A image using KNN

Classified classes	Reference classes									
	Agriculture	Sparse vegetation	Barren	Scrub	Juniper patch	Settlement	Permafrost	Water	Road	Total
Agriculture	56	1	0	3	4	38	0	0	6	108
Sparse vegetation	7	64	0	4	8	0	0	1	0	84
Barren	1	3	90	4	4	6	0	10	41	159
Scrub	7	19	0	78	2	2	0	0	2	110
Juniper patch	17	3	0	1	72	16	0	1	23	133
Settlement	1	0	0	0	0	25	0	0	1	27
Permafrost	0	0	0	0	0	0	90	1	0	91
Water	0	0	0	0	0	0	0	77	0	77
Road	1	0	0	0	0	3	0	0	17	21
Total	90	90	90	90	90	90	90	90	90	810

Overall accuracy (OA): 70.24; Kappa coefficients (κ): 0.67; SE of kappa: 0.02; Weighted Kappa= 0.60

95% confidence interval: From 0.63 to 0.70

Conclusion: The level of agreement is considered as 'Substantial'

(I)

Classification of the Sentinel 2A image using SVM

Classified classes	Reference classes									
	Agriculture	Sparse vegetation	Barren	Scrub	Juniper patch	Settlement	Permafrost	Water	Road	Total
Agriculture	44	7	0	13	8	10	0	0	1	83
Sparse vegetation	1	59	1	3	5	0	0	0	0	69
Barren	1	4	84	0	0	1	4	3	9	106
Scrub	2	13	4	71	1	0	0	0	0	91
Juniper patch	8	5	0	1	67	4	0	0	7	92
Settlement	31	1	0	0	3	63	0	0	9	107
Permafrost	0	0	0	0	0	0	86	0	0	86
Water	0	0	1	0	0	1	0	83	1	86
Road	3	1	0	2	6	11	0	4	63	90
Total	90	90	90	90	90	90	90	90	90	810

Overall accuracy (OA): 76.54; Kappa coefficients (κ): 0.74; SE of kappa: 0.02; Weighted Kappa= 0.72

95% confidence interval: From 0.70 to 0.77

Conclusion: The level of agreement is considered as 'Substantial'

(J)

Classified classes	Agriculture	Sparse vegetation	Barren	Scrub	Juniper patch	Settlement	Permafrost	Water	Road	Total
	**Classification of the Sentinel 2A image using RF**									
	**Reference classes**									
Agriculture	69	1	0	1	3	16	0	0	4	94
Sparse vegetation	3	67	1	2	8	0	0	0	0	81
Barren	0	5	88	2	4	3	0	10	29	141
Scrub	4	14	1	84	0	0	0	0	2	105
Juniper patch	12	3	0	1	75	6	0	0	15	112
Settlement	1	0	0	0	0	59	0	0	1	61
Permafrost	0	0	0	0	0	0	90	0	1	91
Water	0	0	0	0	0	1	0	80	0	81
Road	1	0	0	0	0	5	0	0	38	44
Total	90	90	90	90	90	90	90	90	90	810

Overall accuracy (OA): 80.24; Kappa coefficients (κ): 0.78; SE of kappa: 0.02; Weighted Kappa= 0.74

95% confidence interval: From 0.75to 0.81

Conclusion: The level of agreement is considered as 'Substantial'

(K)

Classification of the Integrated image using ML

Classified classes	**Reference classes**									
	Agriculture	Sparse vegetation	Barren	Scrub	Juniper patch	Settlement	Permafrost	Water	Road	Total
Agriculture	59	7	0	1	3	14	0	0	2	86
Sparse vegetation	4	40	6	18	4	0	0	0	1	73
Barren	0	5	75	13	3	5	0	2	16	119
Scrub	5	21	6	56	5	0	0	0	0	93
Juniper patch	7	16	0	2	70	1	0	0	9	105
Settlement	13	0	1	0	0	53	0	1	6	74
Permafrost	0	0	0	0	0	0	90	2	0	92
Water	0	0	0	0	0	0	0	83	1	84
Road	2	1	2	0	5	17	0	2	55	84
Total	90	90	90	90	90	90	90	90	90	810

Overall accuracy (OA): 71.73; Kappa coefficients (κ): 0.68; SE of kappa: 0.02; Weighted Kappa= 0.71

95% confidence interval: From 0.65 to 0.72

Conclusion: The level of agreement is considered as 'Substantial'

(L)

		Classification of the Integrated image using GMM									
		**Reference classes**									
		Agriculture	Sparse vegetation	Barren	Scrub	Juniper patch	Settlement	Permafrost	Water	Road	Total
**Classified classes**	Agriculture	56	4	0	1	2	22	0	0	4	89
	Sparse vegetation	5	37	1	8	2	0	0	0	1	54
	Barren	2	6	84	18	5	9	0	3	47	174
	Scrub	8	27	5	62	6	0	0	0	0	108
	Juniper patch	8	16	0	1	75	3	0	0	11	114
	Settlement	10	0	0	0	0	42	0	1	4	57
	Permafrost	0	0	0	0	0	0	90	3	0	93
	Water	0	0	0	0	0	0	0	81	1	82
	Road	1	0	0	0	0	14	0	2	22	39
	Total	90	90	90	90	90	90	90	90	90	810
Overall accuracy (OA): 67.78; Kappa coefficients (κ): 0.64; SE of kappa: 0.02; Weighted Kappa= 0.61											
95% confidence interval: From 0.60 to 0.67											
Conclusion: The level of agreement is considered as 'Substantial'											

(M)

Classification of the Integrated image using KNN

Classified classes	Reference classes									
	Agriculture	Sparse vegetation	Barren	Scrub	Juniper patch	Settlement	Permafrost	Water	Road	Total
Agriculture	69	2	0	6	3	14	0	0	3	97
Sparse vegetation	1	72	1	3	6	0	0	0	1	84
Barren	0	2	86	7	4	4	0	3	10	116
Scrub	5	12	3	71	2	2	0	1	1	97
Juniper patch	7	2	0	3	73	7	0	0	9	101
Settlement	6	0	0	0	0	56	0	0	4	66
Permafrost	0	0	0	0	0	0	90	0	0	90
Water	0	0	0	0	0	1	0	86	2	89
Road	2	0	0	0	2	6	0	0	60	70
Total	90	90	90	90	90	90	90	90	90	810

Overall accuracy (OA): 81.85; Kappa coefficients (κ): 0.80; SE of kappa: 0.01; Weighted Kappa= 0.79

95% confidence interval: From 0.77 to 0.83

Conclusion: The level of agreement is considered as 'Substantial'

(N)

Classified classes	Classification of the Integrated image using SVM — Reference classes: Agriculture	Sparse vegetation	Barren	Scrub	Juniper patch	Settlement	Permafrost	Water	Road	Total
Agriculture	47	7	0	11	6	12	0	0	1	84
Sparse vegetation	2	67	6	5	6	0	0	0	0	86
Barren	1	3	73	2	1	0	0	1	4	85
Scrub	2	9	4	68	1	0	0	0	1	85
Juniper patch	7	4	1	3	66	6	0	0	7	94
Settlement	30	0	0	0	2	59	0	0	7	98
Permafrost	0	0	0	0	0	1	90	3	0	94
Water	0	0	1	0	0	0	0	84	2	87
Road	1	0	5	1	8	12	0	2	68	97
Total	90	90	90	90	90	90	90	90	90	810
Overall accuracy (OA): 76.79; Kappa coefficients (κ): 0.74; SE of kappa: 0.02; Weighted Kappa= 0.74										
95% confidence interval: From 0.71 to 0.77										
Conclusion: The level of agreement is considered as 'Substantial'.										

(O)

Classification of the Integrated image using RF

Classified classes	Agriculture	Sparse vegetation	Barren	Scrub	Juniper patch	Settlement	Permafrost	Water	Road	Total
	**Reference classes**									
Agriculture	77	1	0	2	2	9	0	0	1	92
Sparse vegetation	1	72	1	1	4	1	0	0	1	81
Barren	1	4	89	12	3	6	0	2	16	133
Scrub	4	11	0	74	3	2	0	0	1	95
Juniper patch	5	2	0	1	78	1	0	0	7	94
Settlement	2	0	0	0	0	69	0	0	1	72
Permafrost	0	0	0	0	0	0	90	0	0	90
Water	0	0	0	0	0	0	0	87	1	88
Road	0	0	0	0	0	2	0	1	62	65
Total	90	90	90	90	90	90	90	90	90	810

Overall accuracy (OA): 86.17; Kappa coefficients (κ): 0.84; SE of kappa: 0.01; Weighted Kappa= 0.84

95% confidence interval: From 0.82 to 0.87

Conclusion: The level of agreement is considered as 'Almost perfect'
