# Supplementary material for: Beyond traditional methods: Innovative integration of LISS IV and Sentinel 2A imagery for unparalleled insight into Himalayan ibex habitat suitability
Source: PLoS One. 2024 Oct 21;19(10):e0306917. doi: 10.1371/journal.pone.0306917 (PMC11493286; doi:10.1371/journal.pone.0306917)
Supplement: S2 Table — (PDF) [file pone.0306917.s002.pdf]

S2 Table. Independent variables utilised to forecast Himalayan ibex ensemble distribution in Jispa valley landscape using three different sourced images. The correlation test used 19 independent predictors from three key categories: land cover land use classes, radiometric Indices, and topographic variables. The chosen variables for the ensemble modelling are denoted by '#, '*', '$'. '#' denotes variables used in LISS IV derived LCLU SDM, with other variables, '*' denotes variables used in Sentinel 2A derived LCLU SDM, with other variables, '$' denotes variables used in integrated image derived LCLU SDM, with other variables.

Data type	Variable name	Code
Land Cover Land Use classes	Euclidian distance from agriculture	agriculture
	Euclidian distance from sparse vegetation	sparse_veg*$
	Euclidian distance from barren	barren#*$
	Euclidian distance from juniper patch	juniper_patch#*$
	Euclidian distance from road	road
	Euclidian distance from scrub	scrub#*$
	Euclidian distance from settlement	settlement#*$
	Euclidian distance from water	water#*$
Radiometric Indices	Brightness Index	bi
	Colour Index	ci#*$
	Normalized Difference Moisture Index	ndmi#*$
	Normalized Difference Vegetation Index	ndvi
	Soil Adjusted Vegetation Index	savi#*$
Topographical variables	Elevation	dem#*$
	Terrain ruggedness	ruggedness#*$
	Heat Load Index	hli
	Slope	slope#*$
	Aspect	aspect#*$
	Compound Topographic Index	cti
