# Supplementary material for: Beyond traditional methods: Innovative integration of LISS IV and Sentinel 2A imagery for unparalleled insight into Himalayan ibex habitat suitability
Source: PLoS One. 2024 Oct 21;19(10):e0306917. doi: 10.1371/journal.pone.0306917 (PMC11493286; doi:10.1371/journal.pone.0306917)
Supplement: S1 Fig — (PDF) [file pone.0306917.s003.pdf]

S1 Fig. Correlation matrix among the all variables and final variable selection based on <0.8 for the predicting final species distribution models of Himalayan ibex. The distribution of the predictors and the locations of presence and absence indicated by red and blue dots, respectively. The respective predictor shown on the response curve and in the percentage of deviation in the matrix on the left is indicated. (A) Best classified (SVM) LISS IV derived LCLU classes used for this model building with other topographic and radiometric variables, (B) Best classified (RF) Sentinel 2A derived LCLU classes used for this model building with other topographic and radiometric variables. (C) Best classified (RF) Integrated image derived LCLU classes used for this model building with other topographic and radiometric variables.

(A)

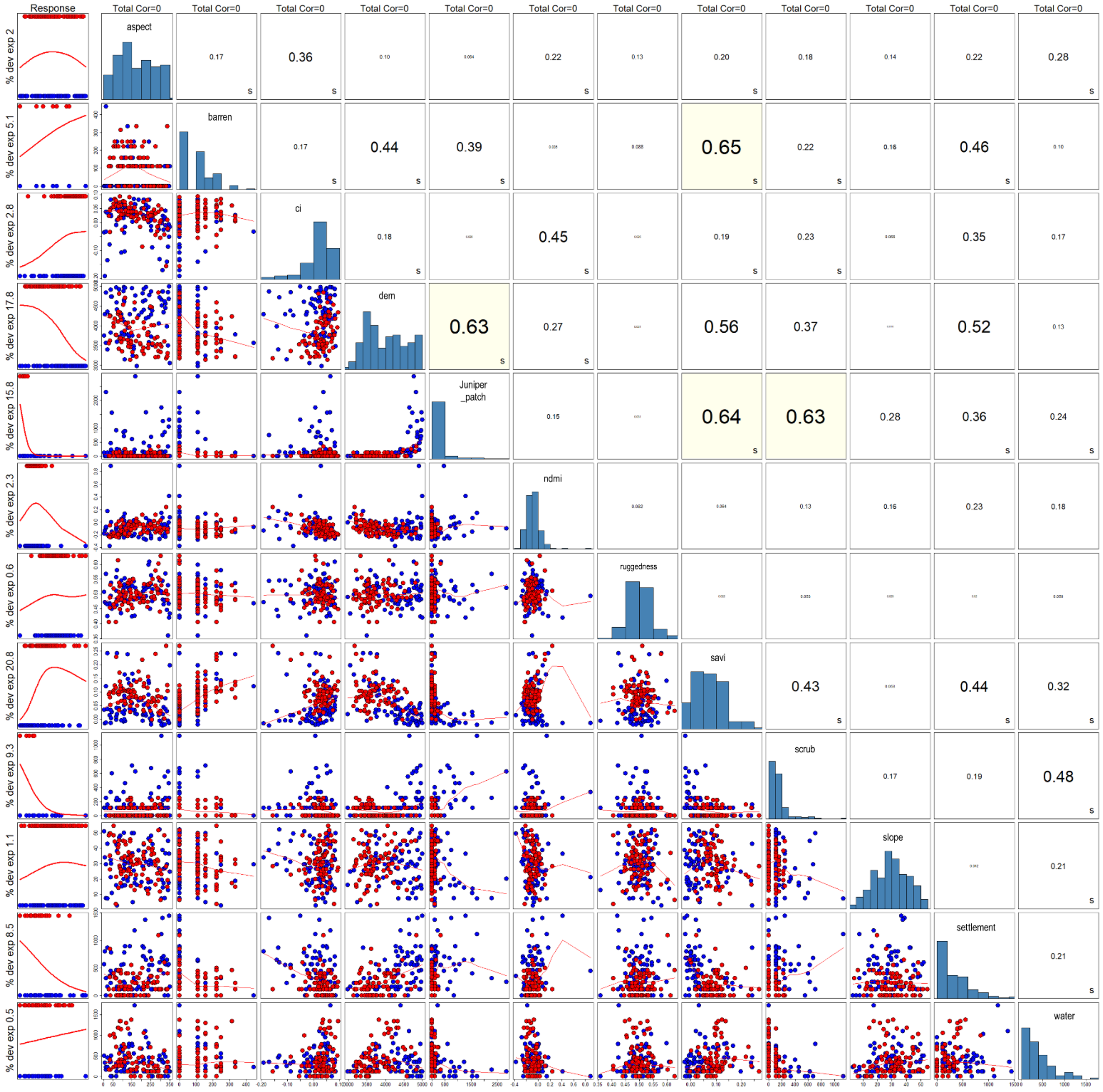

(B)

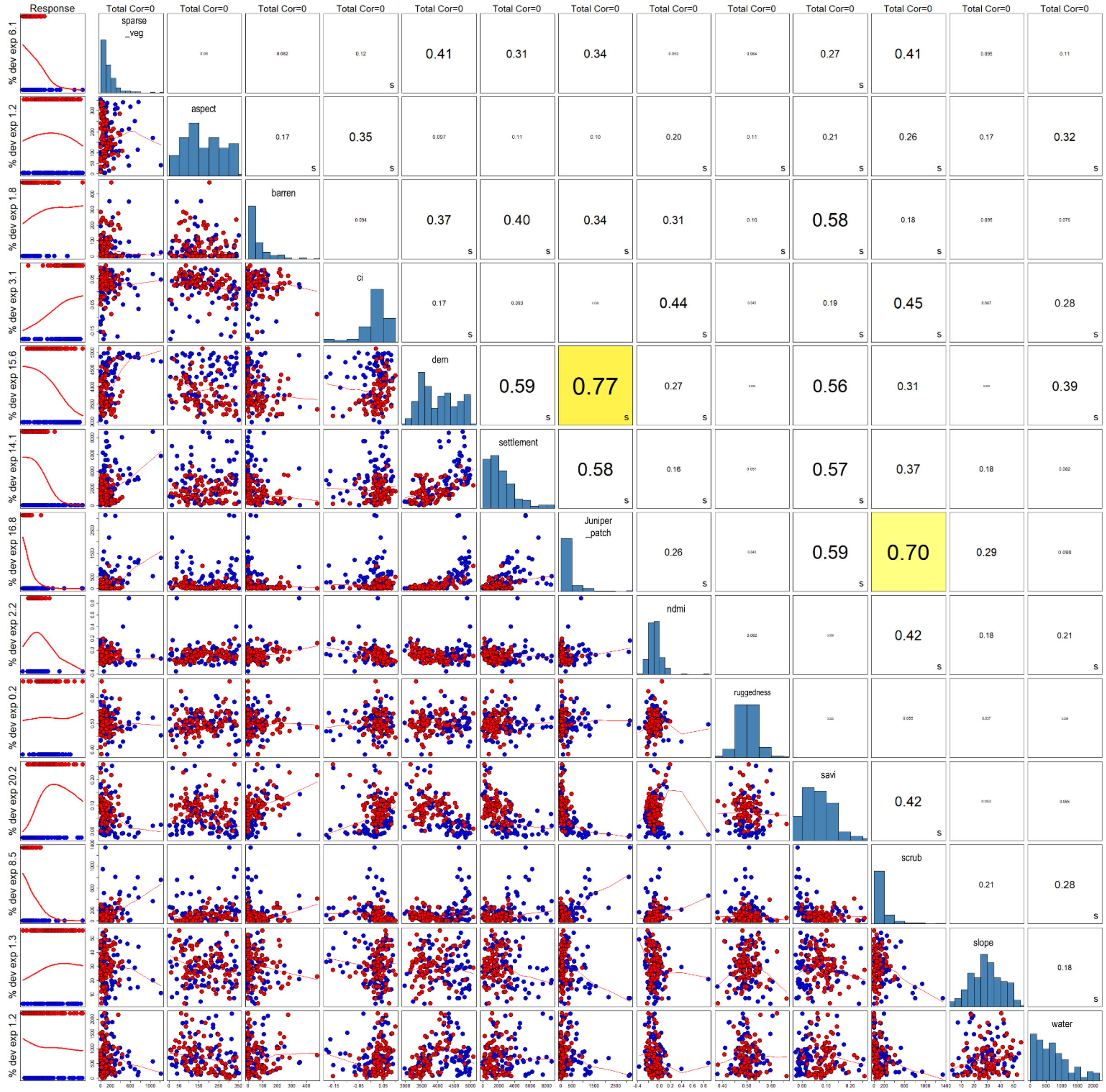

Response
Total Cor=0
% dev exp 6.1
% dev exp 1.2
% dev exp 1.8
% dev exp 3.1
% dev exp 15.6
% dev exp 14.1
% dev exp 16.8
% dev exp 2.2
% dev exp 0.2
% dev exp 20.2
% dev exp 8.5
% dev exp 1.3
% dev exp 1.2
sparse _veg
0.41
0.31
0.34
0.27
0.41
aspect
0.17
0.35
0.20
0.21
0.26
0.17
0.32
barren
0.37
0.40
0.34
0.31
0.58
0.18
ci
0.17
0.44
0.19
0.45
0.28
dem
0.59
0.77
0.27
0.56
0.31
0.39
settlement
0.58
0.16
0.57
0.37
0.18
Juniper _patch
0.26
0.59
0.70
0.29
ndmi
0.42
0.18
0.21
ruggedness
savi
0.42
scrub
0.21
0.28
slope
0.18
water

(C)

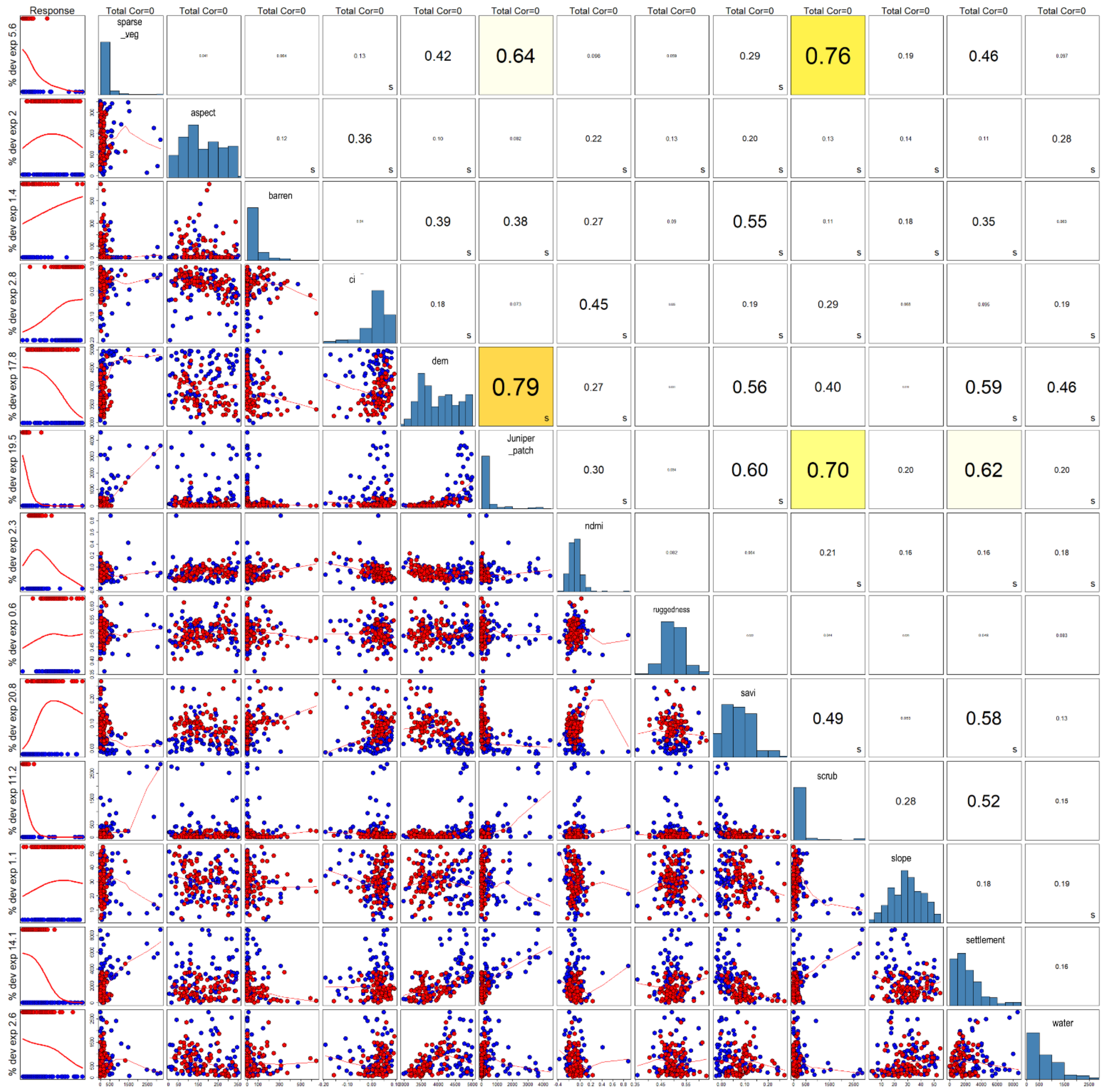

Response
Total Cor=0
sparse_veg
aspect
barren
ci
dem
Juniper_patch
ndmi
ruggedness
savi
scrub
slope
settlement
water
% dev exp 5.6
% dev exp 2
% dev exp 1.4
% dev exp 2.8
% dev exp 17.8
% dev exp 19.5
% dev exp 2.3
% dev exp 0.6
% dev exp 20.8
% dev exp 11.2
% dev exp 1.1
% dev exp 14.1
% dev exp 2.6
0.13
0.42
0.64
0.29
0.76
0.19
0.46
0.12
0.36
0.22
0.13
0.20
0.13
0.14
0.11
0.28
0.39
0.38
0.27
0.55
0.11
0.18
0.35
0.18
0.45
0.19
0.29
0.19
0.79
0.27
0.56
0.40
0.59
0.46
0.30
0.60
0.70
0.20
0.62
0.20
0.21
0.16
0.16
0.18
0.49
0.58
0.13
0.28
0.52
0.15
0.18
0.19
0.16
