# Supplementary material for: Beyond traditional methods: Innovative integration of LISS IV and Sentinel 2A imagery for unparalleled insight into Himalayan ibex habitat suitability
Source: PLoS One. 2024 Oct 21;19(10):e0306917. doi: 10.1371/journal.pone.0306917 (PMC11493286; doi:10.1371/journal.pone.0306917)
Supplement: S2 Fig — The observed vs. predicted results are presented. (PDF) [file pone.0306917.s004.pdf]

S2 Fig. Different models confusion matrix: Represents the confusion matrix for each of the five models. The observed vs. predicted results are presented, and the quantification of specific pair types is shown by the colour ramp from 0% (white) to 100% (red) while using (A) LISS IV classified image, (B) Sentinel 2A classified image, (C) Integrated classified image along with other topographic and radiometric variables.

(A)

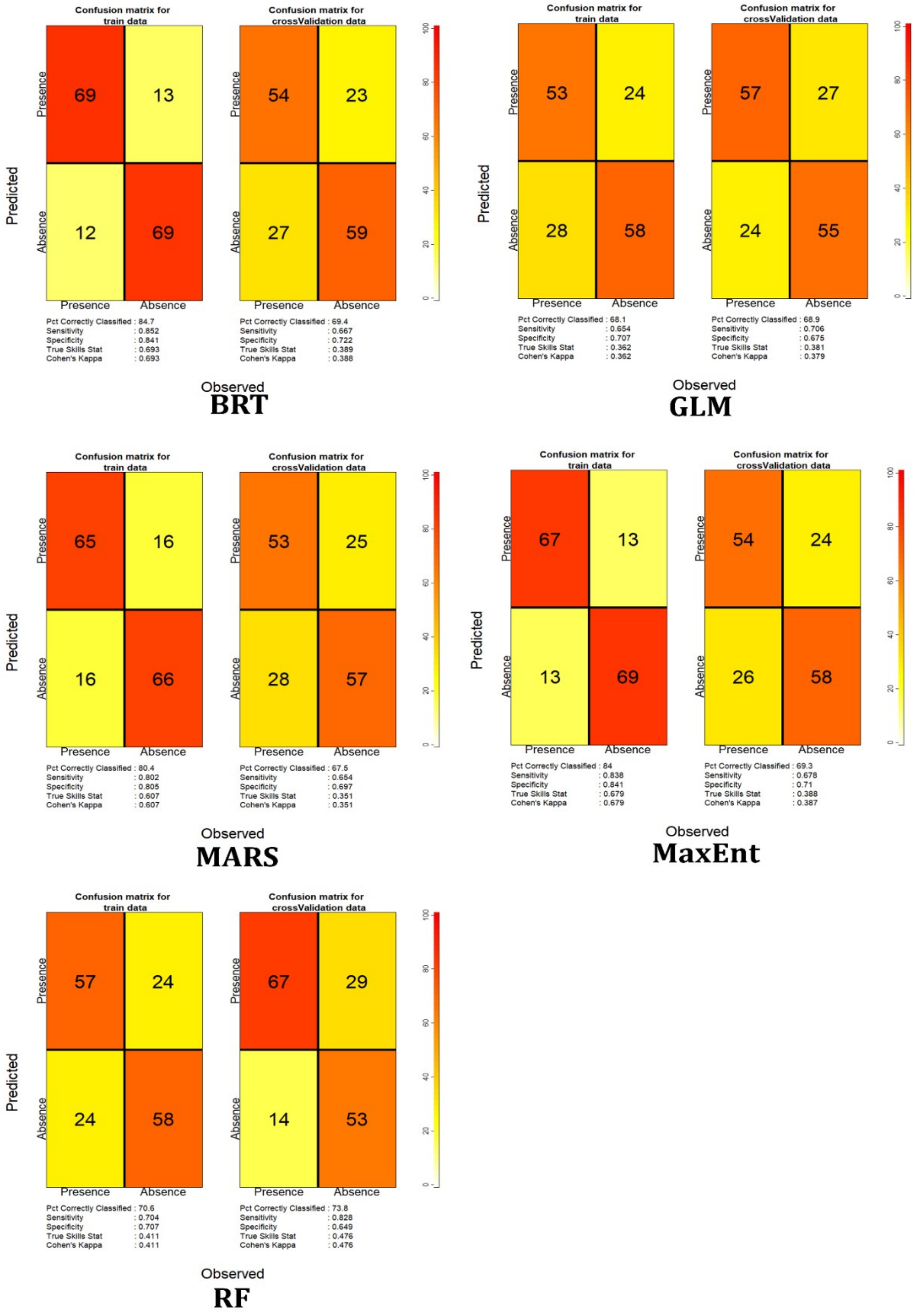

(B)

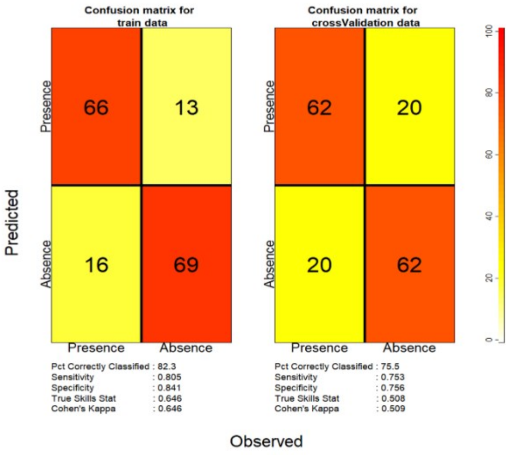

BRT

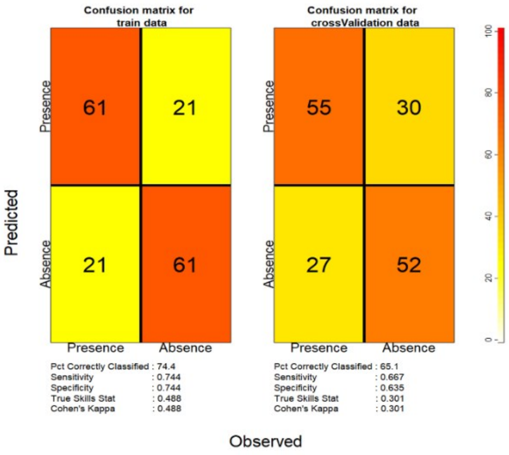

GLM

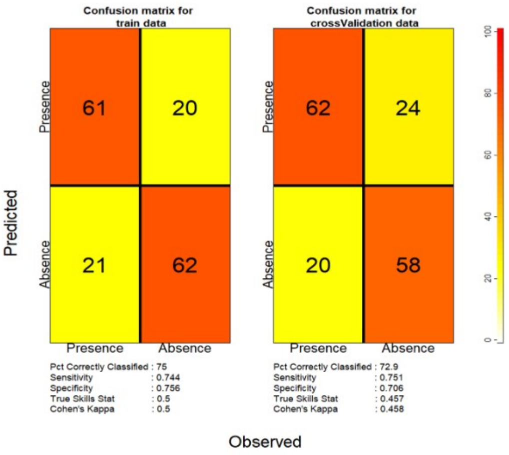

MARS

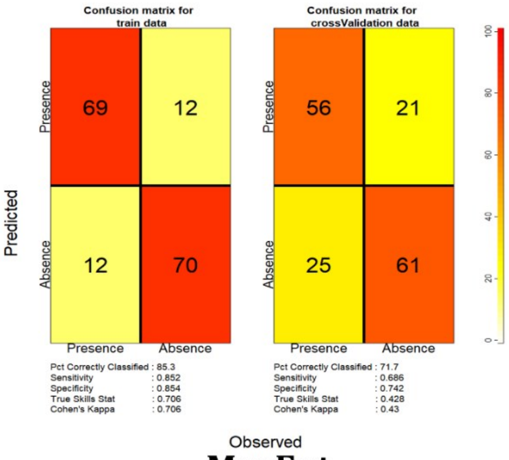

MaxEnt

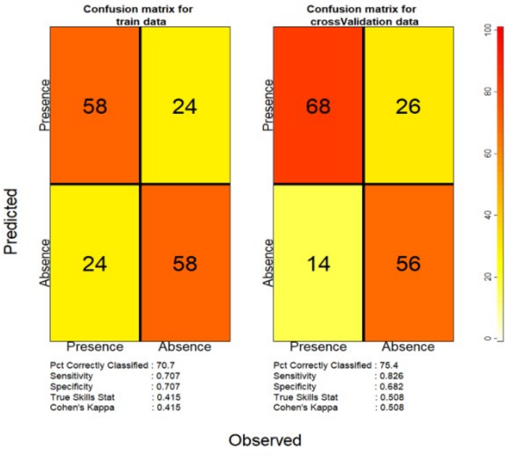

RF

(C)

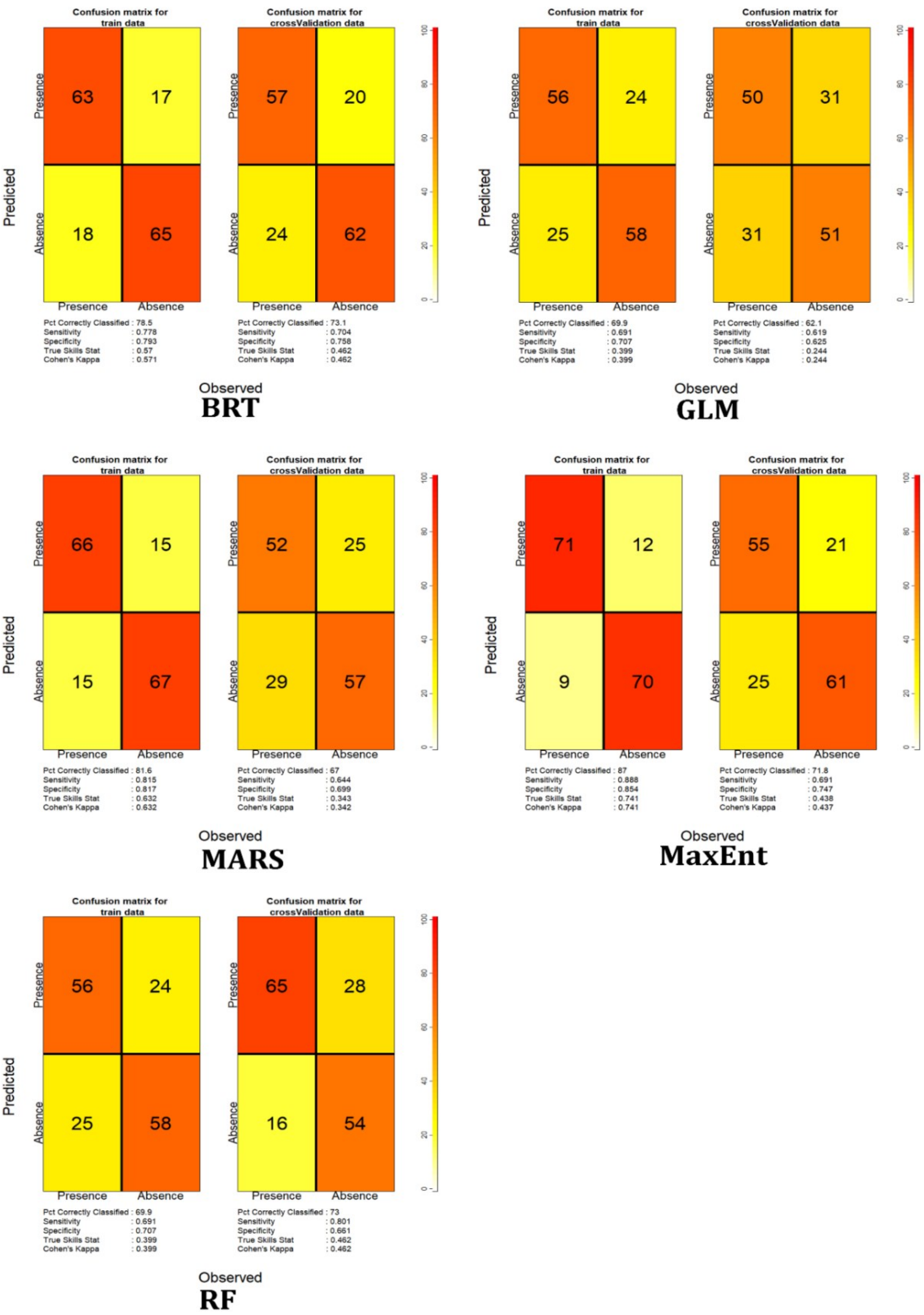
Confusion matrix for train data
Confusion matrix for crossValidation data
Predicted
Presence
Absence
Observed
BRT
63 17 18 65
Pct Correctly Classified : 78.5
Sensitivity : 0.778
Specificity : 0.793
True Skills Stat : 0.57
Cohen's Kappa : 0.571
57 20 24 62
Pct Correctly Classified : 73.1
Sensitivity : 0.704
Specificity : 0.758
True Skills Stat : 0.462
Cohen's Kappa : 0.462
GLM
56 24 25 58
Pct Correctly Classified : 69.9
Sensitivity : 0.691
Specificity : 0.707
True Skills Stat : 0.399
Cohen's Kappa : 0.399
50 31 31 51
Pct Correctly Classified : 62.1
Sensitivity : 0.619
Specificity : 0.625
True Skills Stat : 0.244
Cohen's Kappa : 0.244
MARS
66 15 15 67
Pct Correctly Classified : 81.6
Sensitivity : 0.815
Specificity : 0.817
True Skills Stat : 0.632
Cohen's Kappa : 0.632
52 25 29 57
Pct Correctly Classified : 67
Sensitivity : 0.644
Specificity : 0.699
True Skills Stat : 0.343
Cohen's Kappa : 0.342
MaxEnt
71 12 9 70
Pct Correctly Classified : 87
Sensitivity : 0.888
Specificity : 0.854
True Skills Stat : 0.741
Cohen's Kappa : 0.741
55 21 25 61
Pct Correctly Classified : 71.8
Sensitivity : 0.691
Specificity : 0.747
True Skills Stat : 0.438
Cohen's Kappa : 0.437
RF
56 24 25 58
Pct Correctly Classified : 69.9
Sensitivity : 0.691
Specificity : 0.707
True Skills Stat : 0.399
Cohen's Kappa : 0.399
65 28 16 54
Pct Correctly Classified : 73
Sensitivity : 0.801
Specificity : 0.661
True Skills Stat : 0.462
Cohen's Kappa : 0.462
0 20 40 60 80 100
