# Supplementary material for: Beyond traditional methods: Innovative integration of LISS IV and Sentinel 2A imagery for unparalleled insight into Himalayan ibex habitat suitability
Source: PLoS One. 2024 Oct 21;19(10):e0306917. doi: 10.1371/journal.pone.0306917 (PMC11493286; doi:10.1371/journal.pone.0306917)
Supplement: S3 Fig — (PDF) [file pone.0306917.s005.pdf]

S3 Fig. Calibration plots and residual plots of the five distribution models. On the right column, 5 separate models' calibration plots for the cross-validation division have been illustrated. On the left column the spatial pattern of residuals is showing and the size and colour of the ramp indicate the quantity and magnitude of the deviation. On the right side of the plots, each model's name is displayed. The model plots build with (A) LISS IV classified, (B) Sentinel 2A classified, (C) Integrated image classified LCLU along the topographic and radiometric variables.

(A)

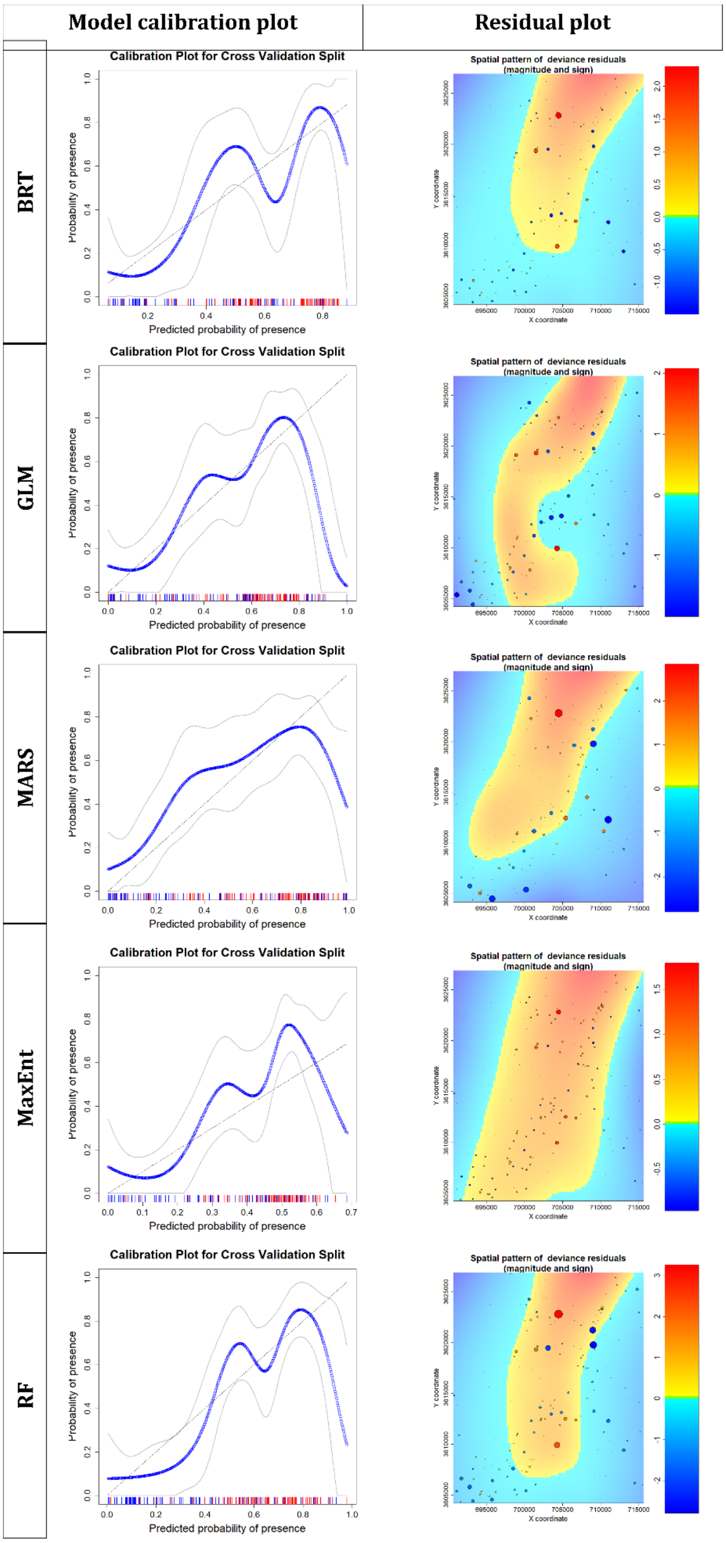

(B)

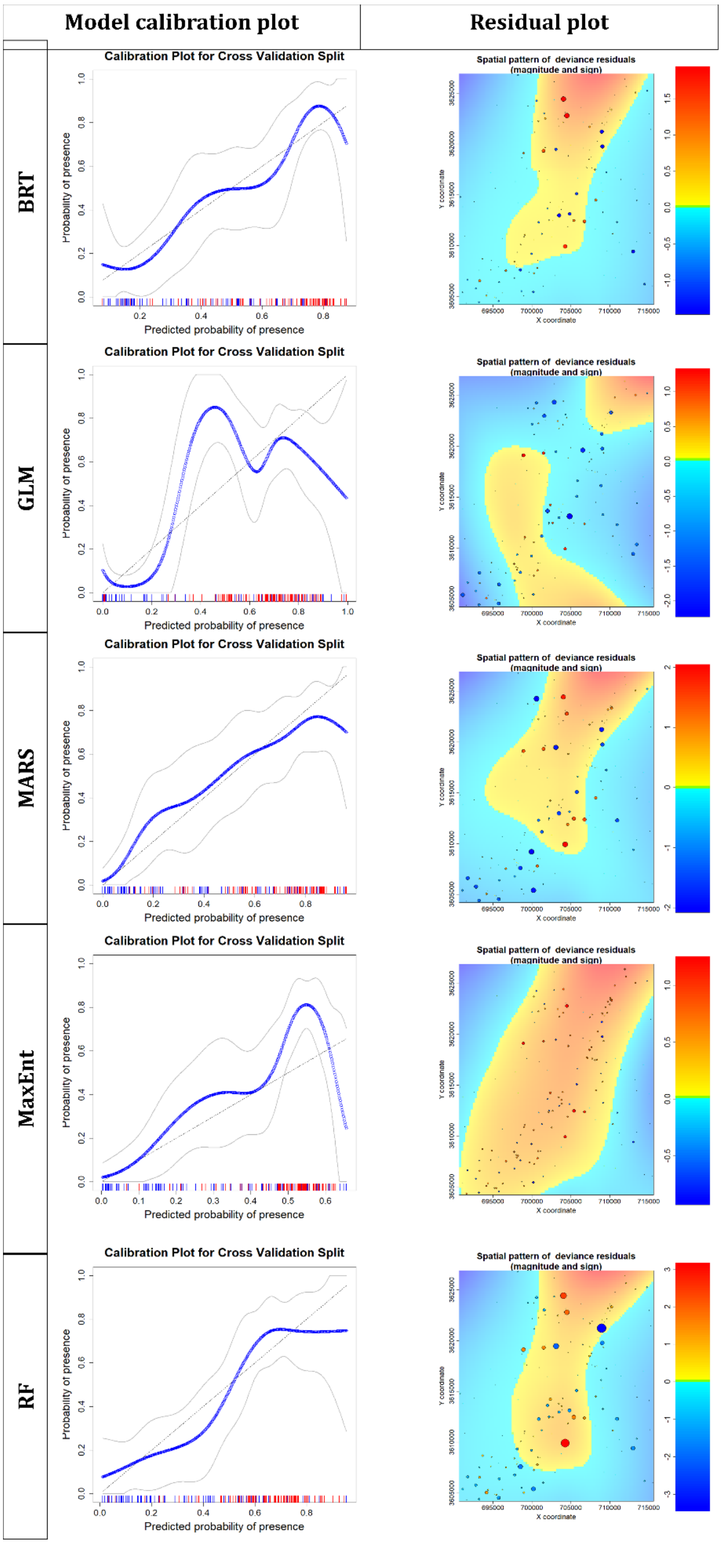

Model calibration plot
Residual plot
BRT
GLM
MARS
MaxEnt
RF
Calibration Plot for Cross Validation Split
Probability of presence
Predicted probability of presence
Spatial pattern of deviance residuals
(magnitude and sign)
X coordinate
Y coordinate

(C)

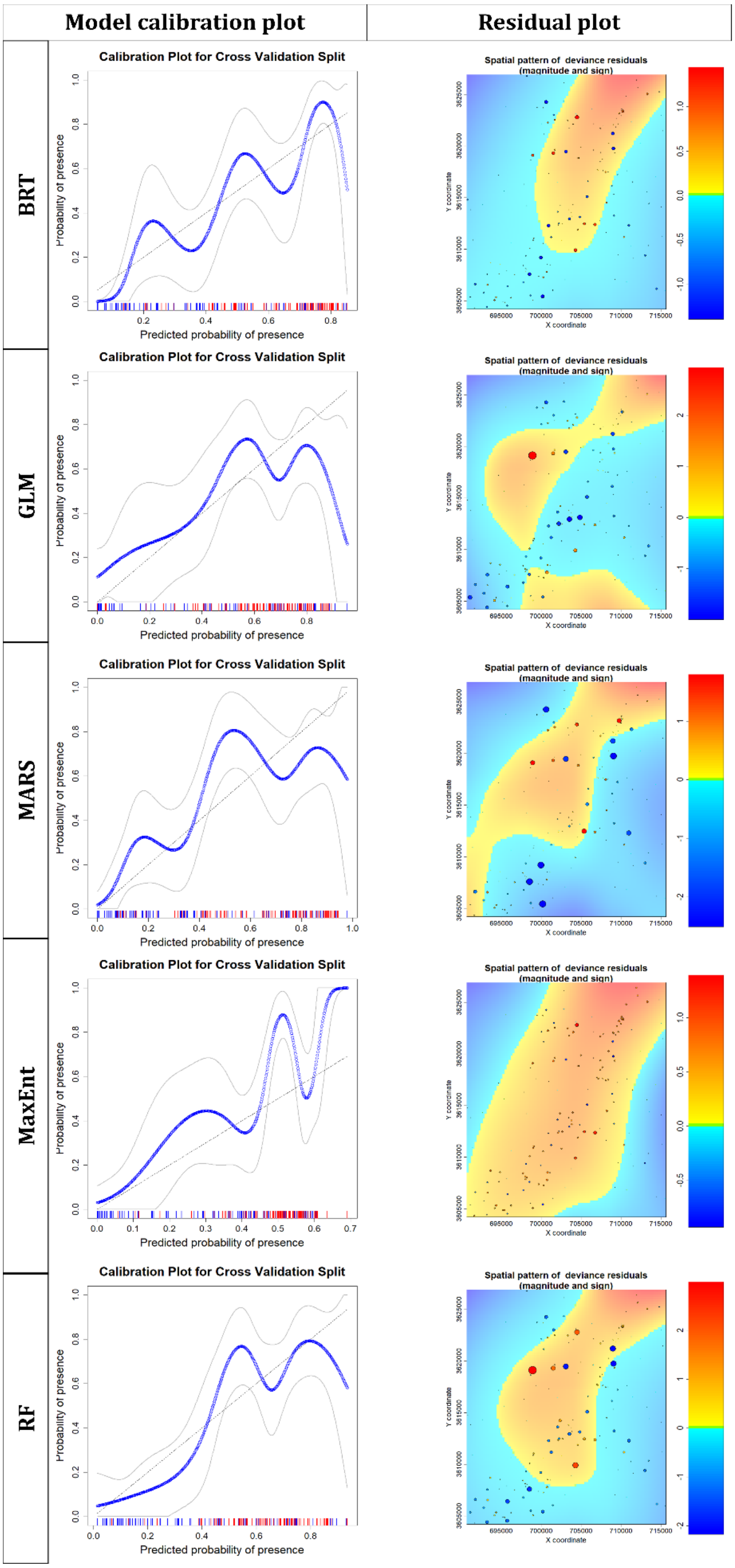
Model calibration plot
Residual plot
BRT
GLM
MARS
MaxEnt
RF
Calibration Plot for Cross Validation Split
Probability of presence
Predicted probability of presence
Spatial pattern of deviance residuals
(magnitude and sign)
Y coordinate
X coordinate
