# Supplementary material for: Beyond traditional methods: Innovative integration of LISS IV and Sentinel 2A imagery for unparalleled insight into Himalayan ibex habitat suitability
Source: PLoS One. 2024 Oct 21;19(10):e0306917. doi: 10.1371/journal.pone.0306917 (PMC11493286; doi:10.1371/journal.pone.0306917)
Supplement: S4 Fig — (PDF) [file pone.0306917.s006.pdf]

S4 Fig. Variable significance: each predictor is permuted for training and cross-validation data, the change in AUC is used to assess the variable's significance, when using the LCLU derived from (A) LISS IV classified image, (B) Sentinel 2A classified image, (C) Integrated image classified image along with other topographic and radiometric variables.

(A)

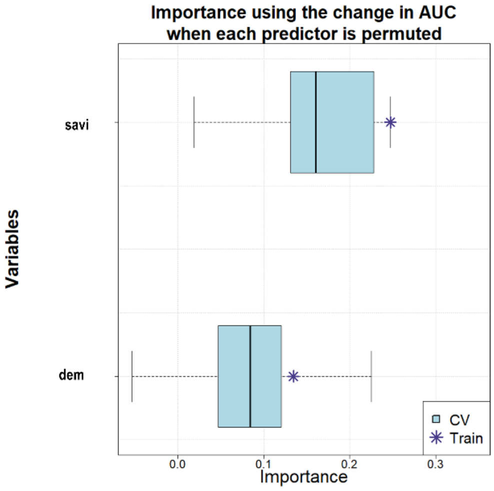

BRT

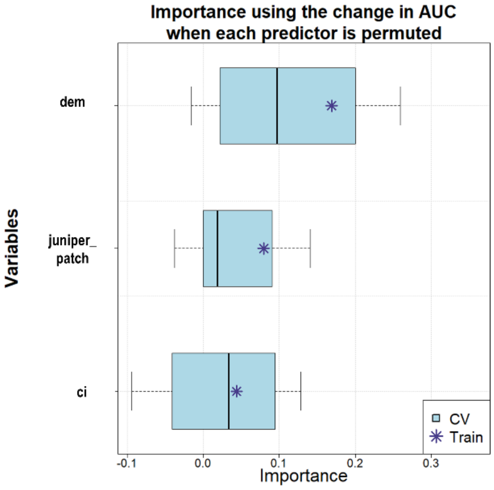

GLM

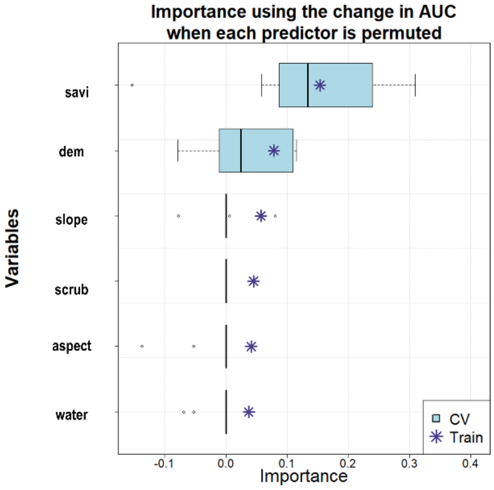

MARS

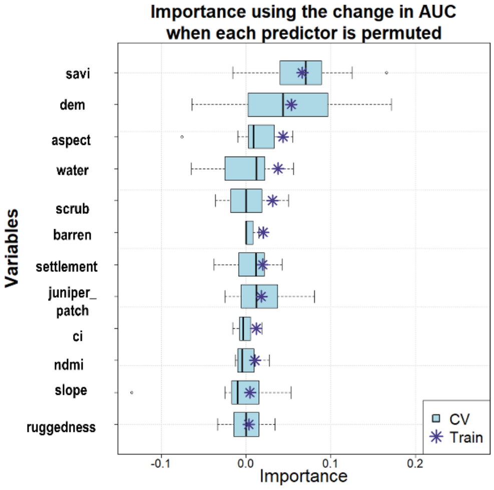

MaxEnt

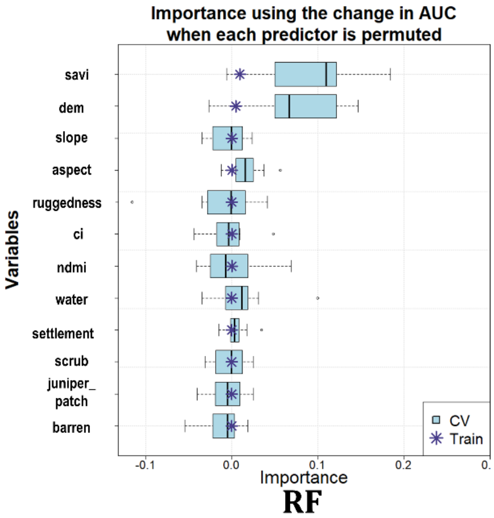

RF

(B)

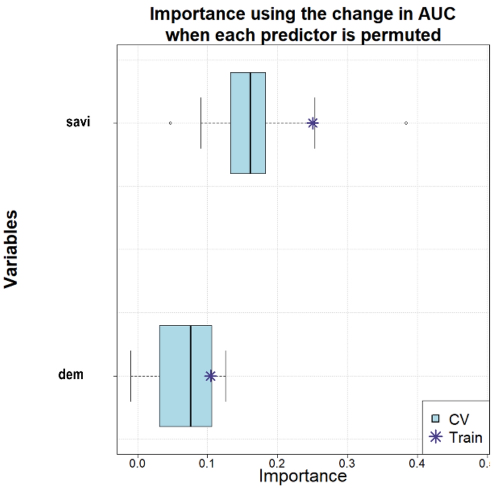

BRT

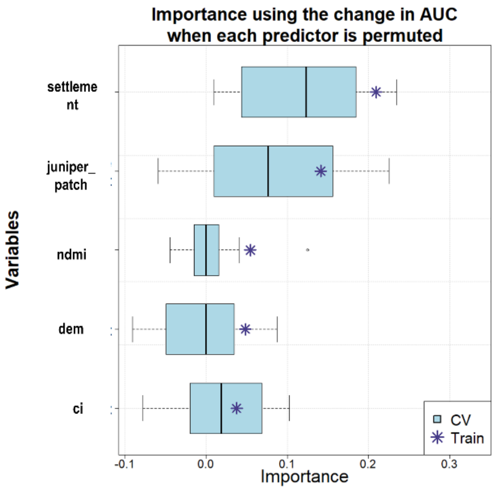

GLM

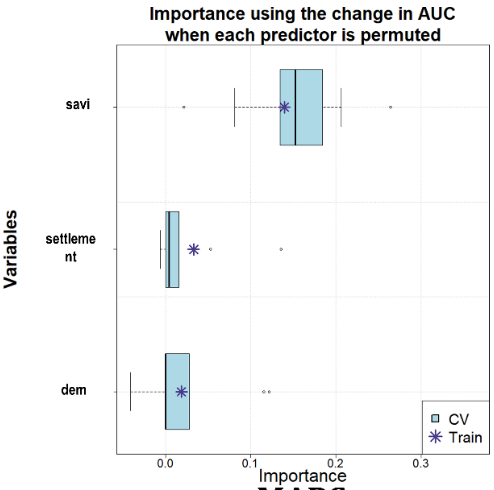

MARS

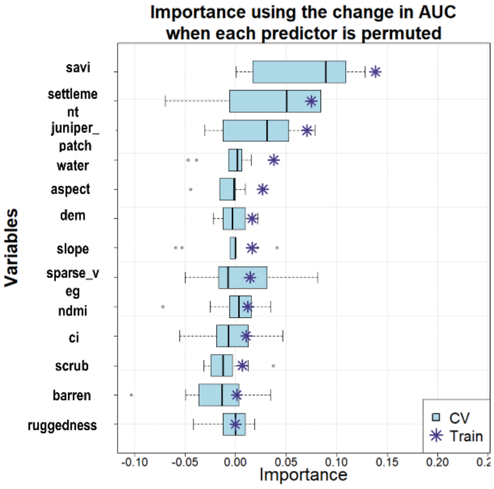

MaxEnt

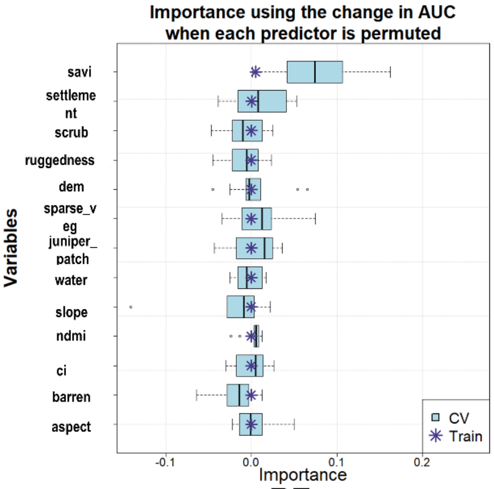

RF

(C)

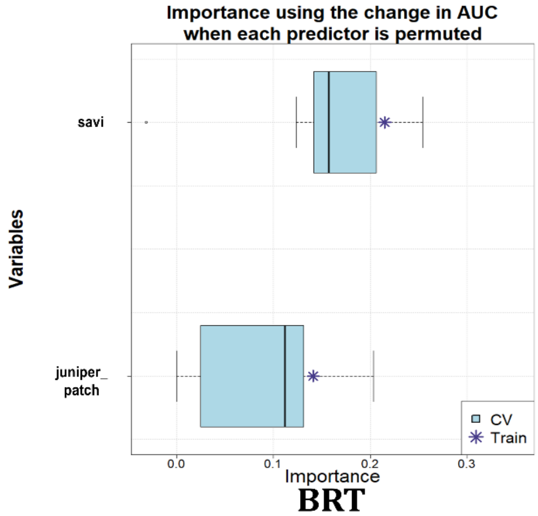

BRT

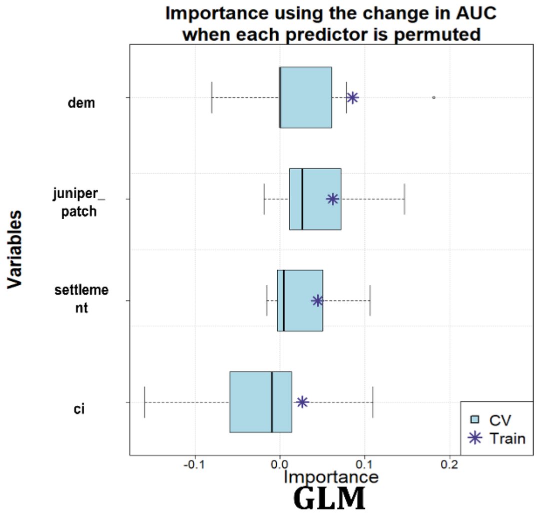

GLM

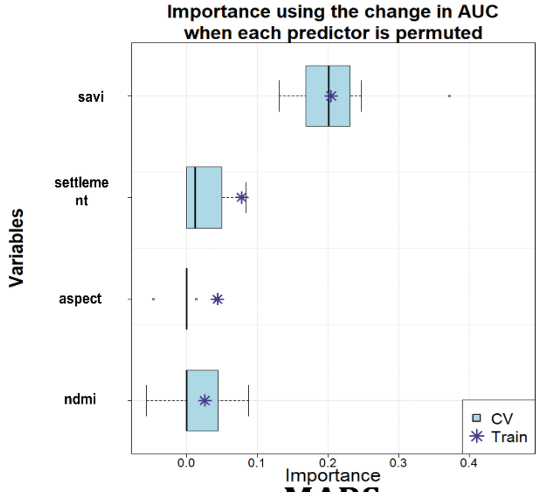

MARS

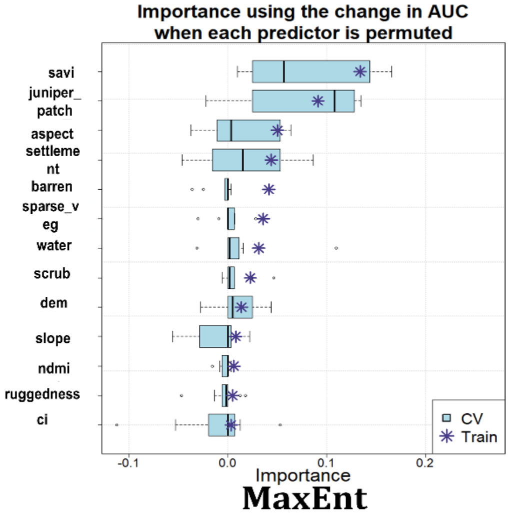

MaxEnt

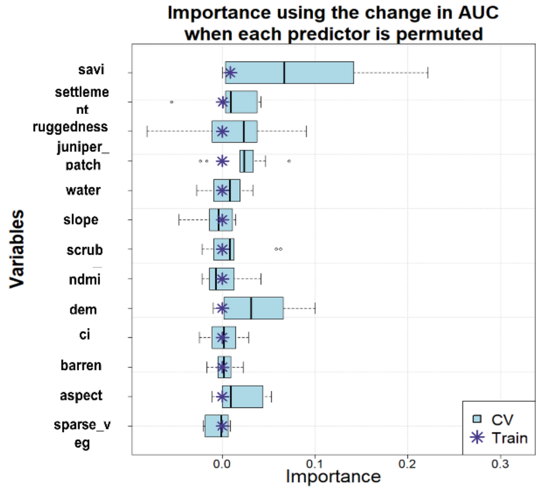

RF
