# Supplementary material for: Beyond traditional methods: Innovative integration of LISS IV and Sentinel 2A imagery for unparalleled insight into Himalayan ibex habitat suitability
Source: PLoS One. 2024 Oct 21;19(10):e0306917. doi: 10.1371/journal.pone.0306917 (PMC11493286; doi:10.1371/journal.pone.0306917)

S5 Fig. Representation of the response curves of the selected variables used by different SDMs. Response curve of (A) BRT, (B) GLM, (C) MARS, (D) MaxEnt, (E) RF model using LISS IV derived LCLU classes and other variables. (F) BRT, (G) GLM, (H) MARS, (I) MaxEnt, (J) RF model using Sentinel 2A derived LCLU classes and other variables. (K) BRT, (L) GLM, (M) MARS, (N) MaxEnt, (O) RF model using Integrated image derived LCLU classes and other variables.

(A) Response curve of variables in BRT model using LISS IV derived LCLU classes and other variables.

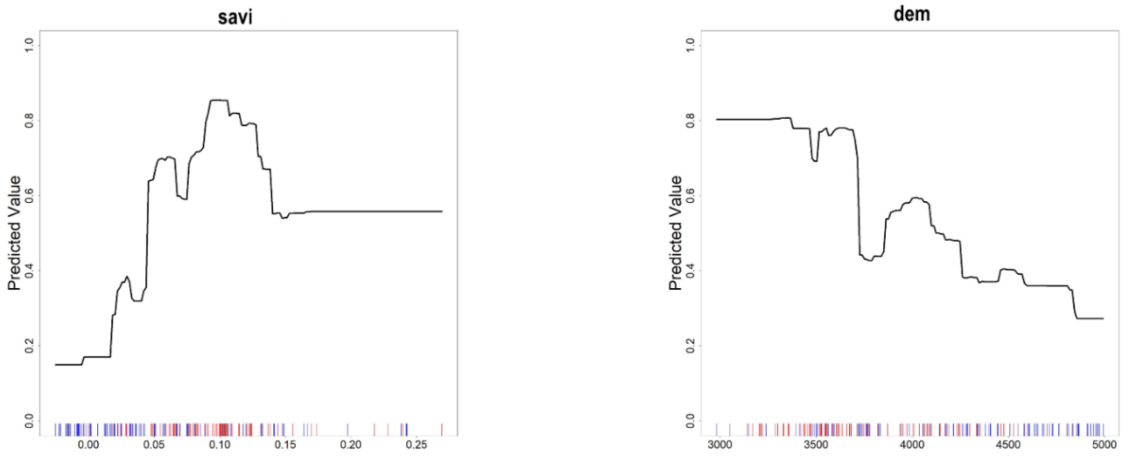

(B) Response curve of variables in GLM model using LISS IV derived LCLU classes and other variables.

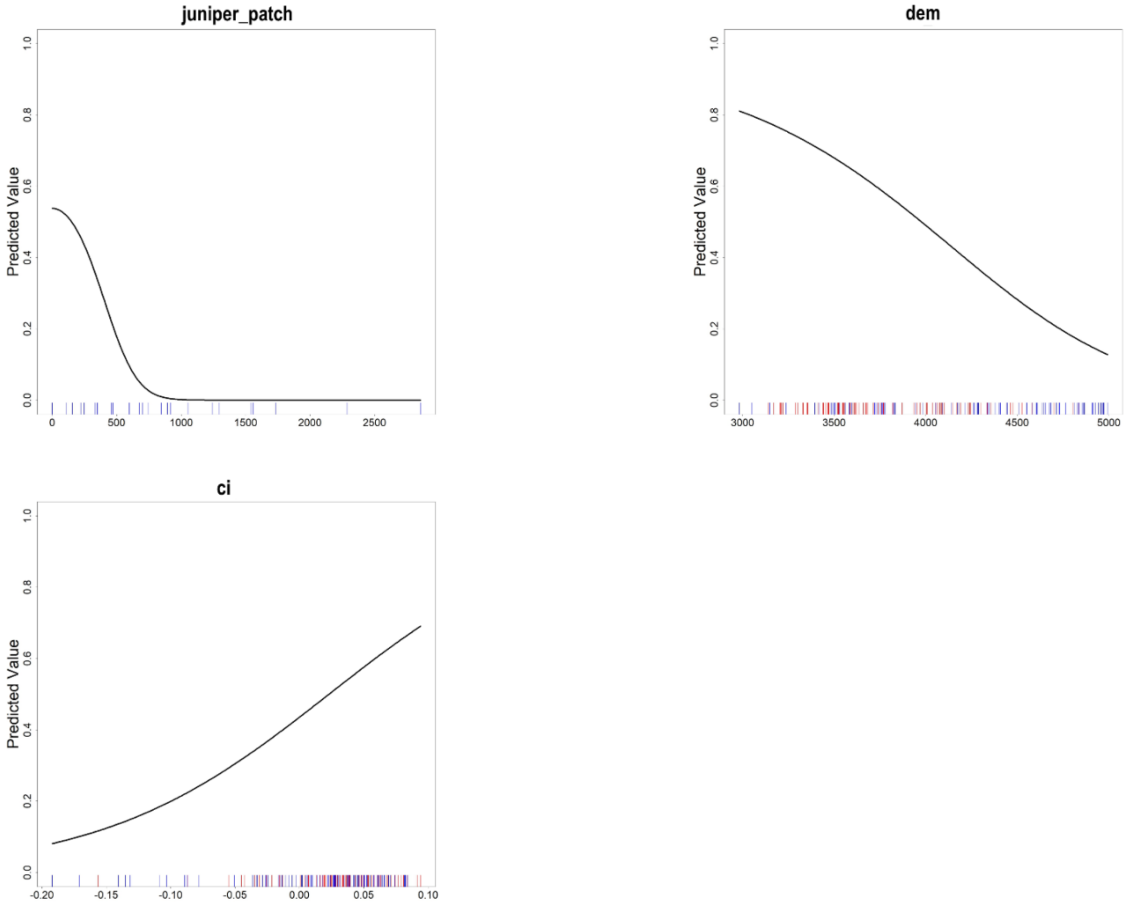

(C) Response curve of variables in MARS model using LISS IV derived LCLU classes and other variables.

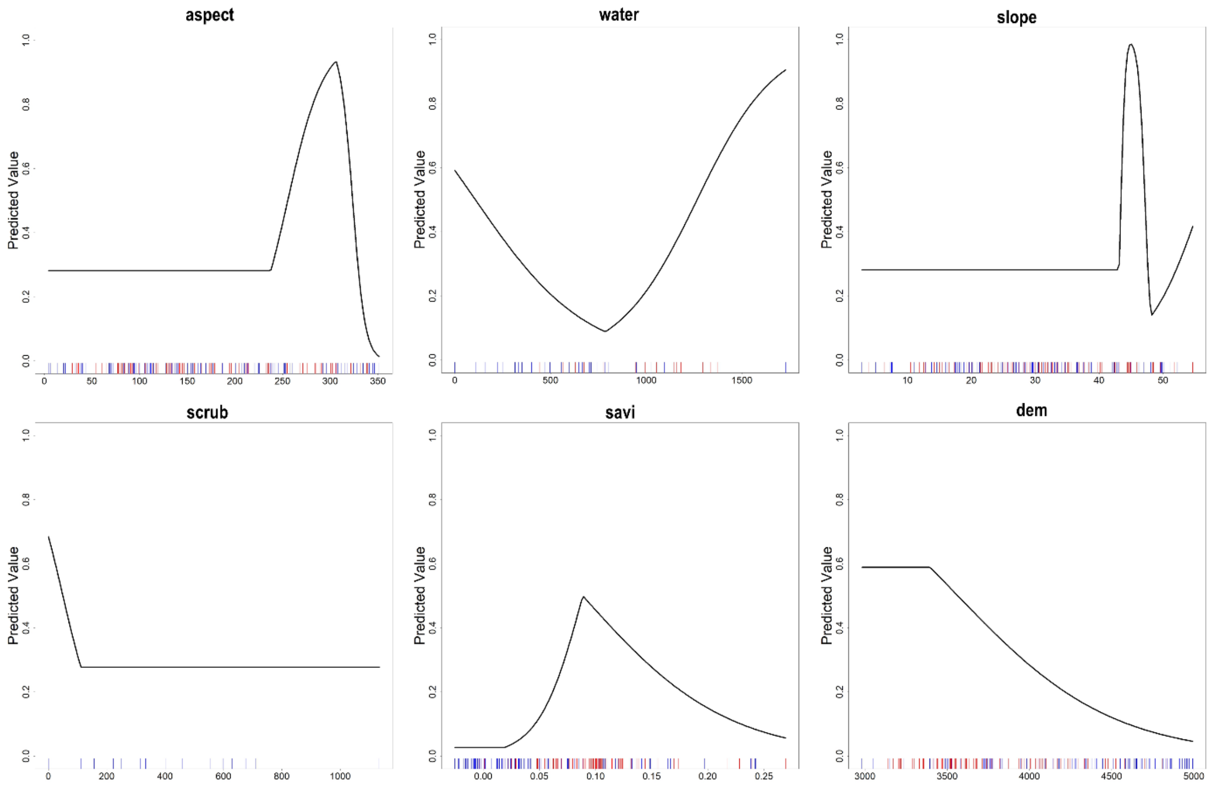

(D) Response curve of variables in MaxEnt model using LISS IV derived LCLU classes and other variables.

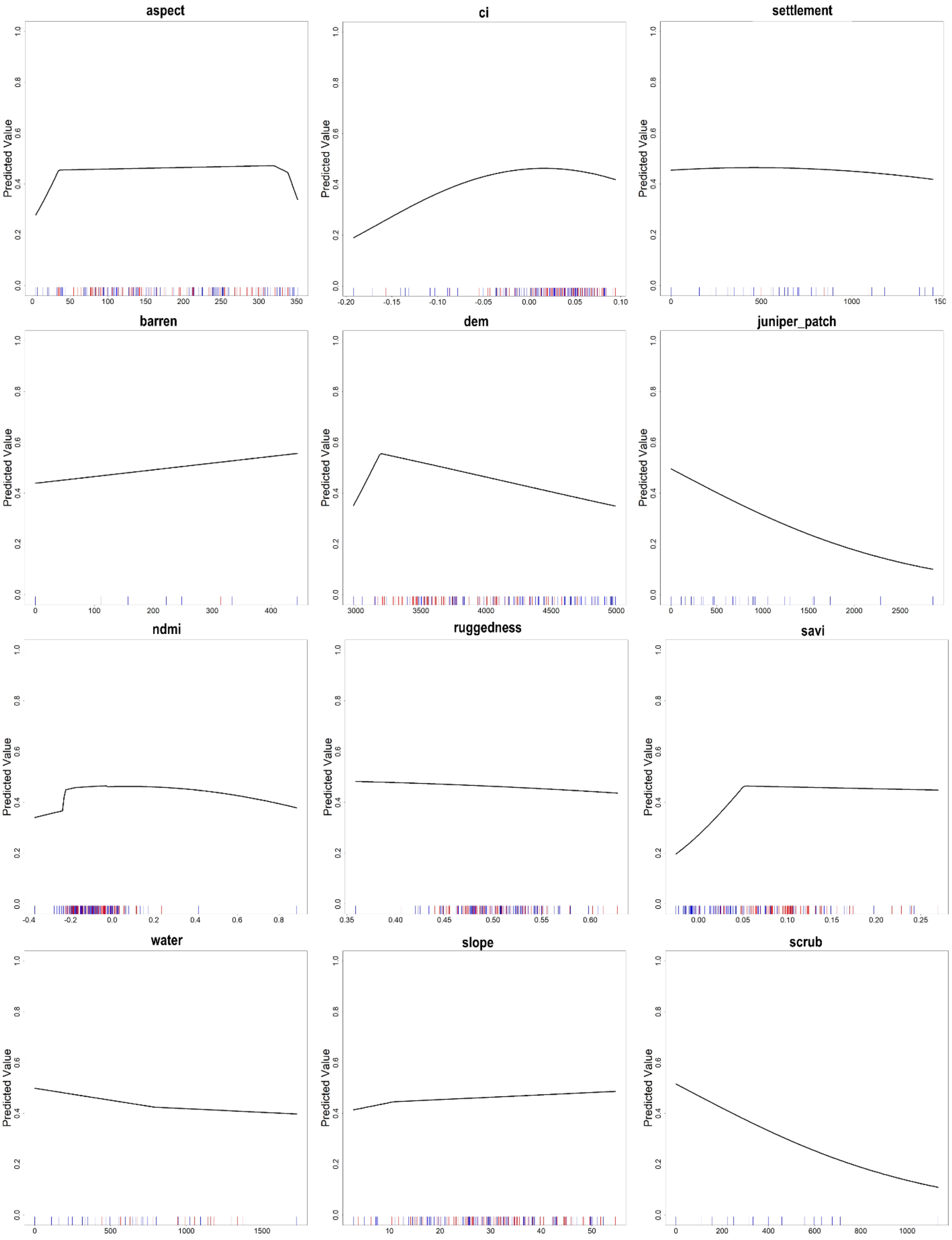

(E) Response curve of variables in RF model using LISS IV derived LCLU classes and other variables.

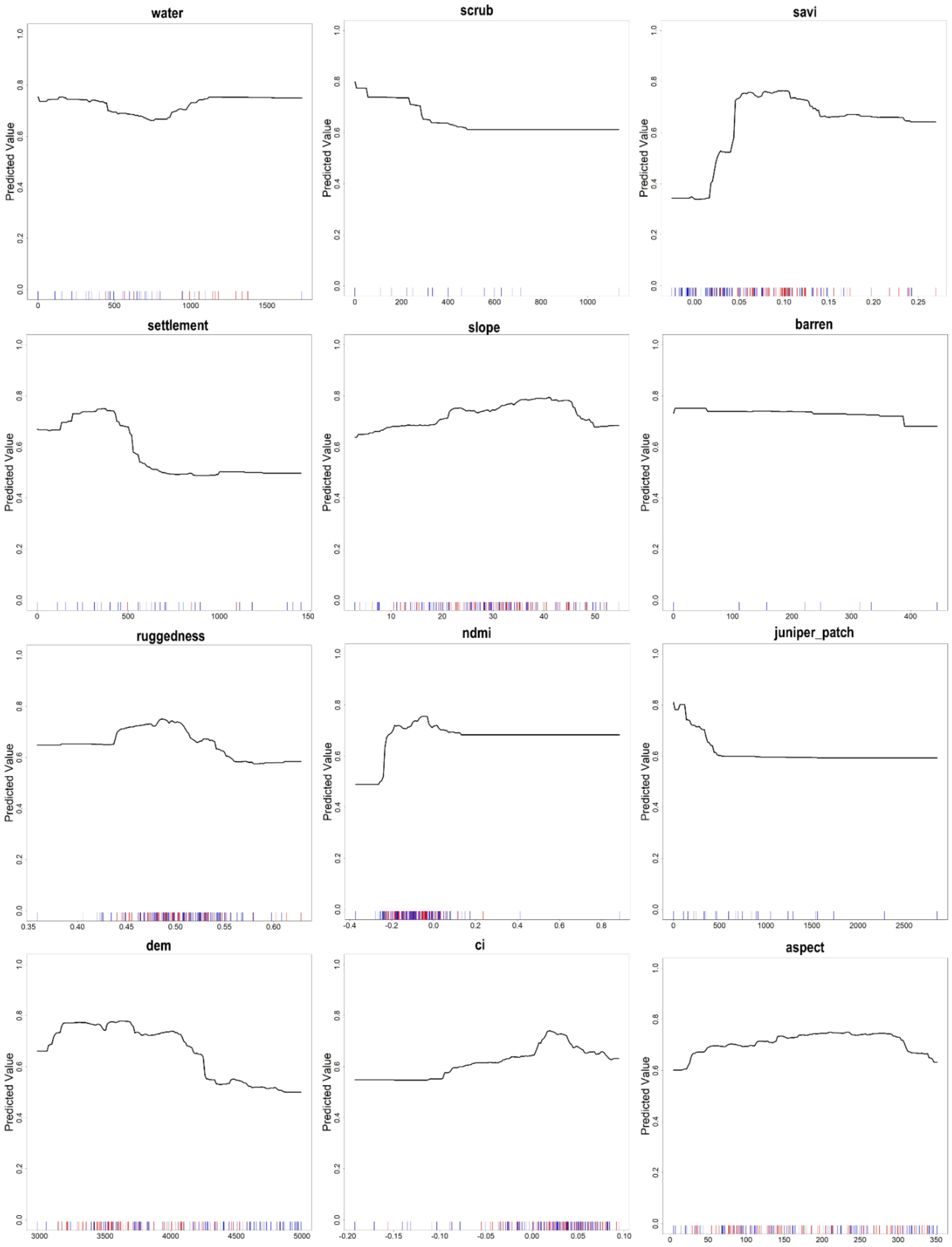

(F) Response curve of variables in BRT model using Sentinel 2A derived LCLU classes and other variables.

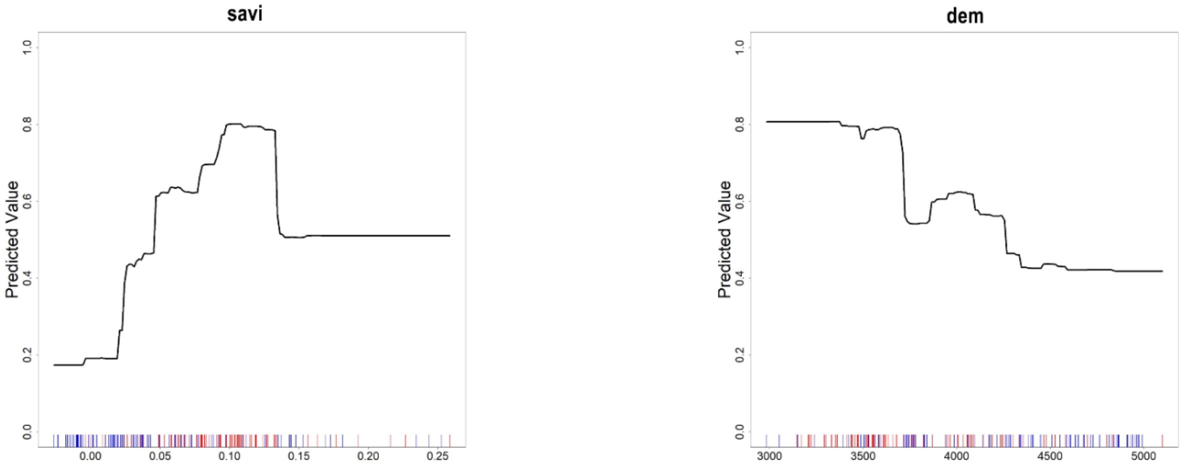

(G) Response curve of variables in GLM model using Sentinel 2A derived LCLU classes and other variables.

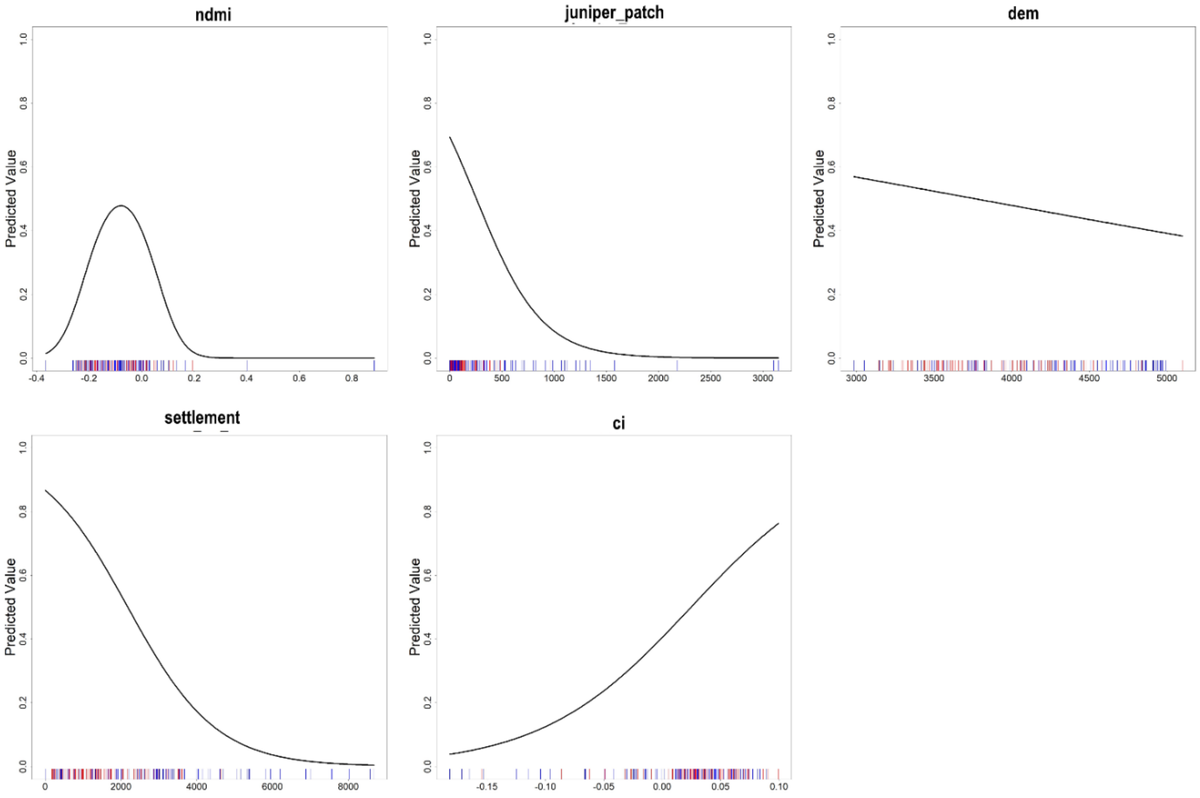

(H) Response curve of variables in MARS model using Sentinel 2A derived LCLU classes and other variables.

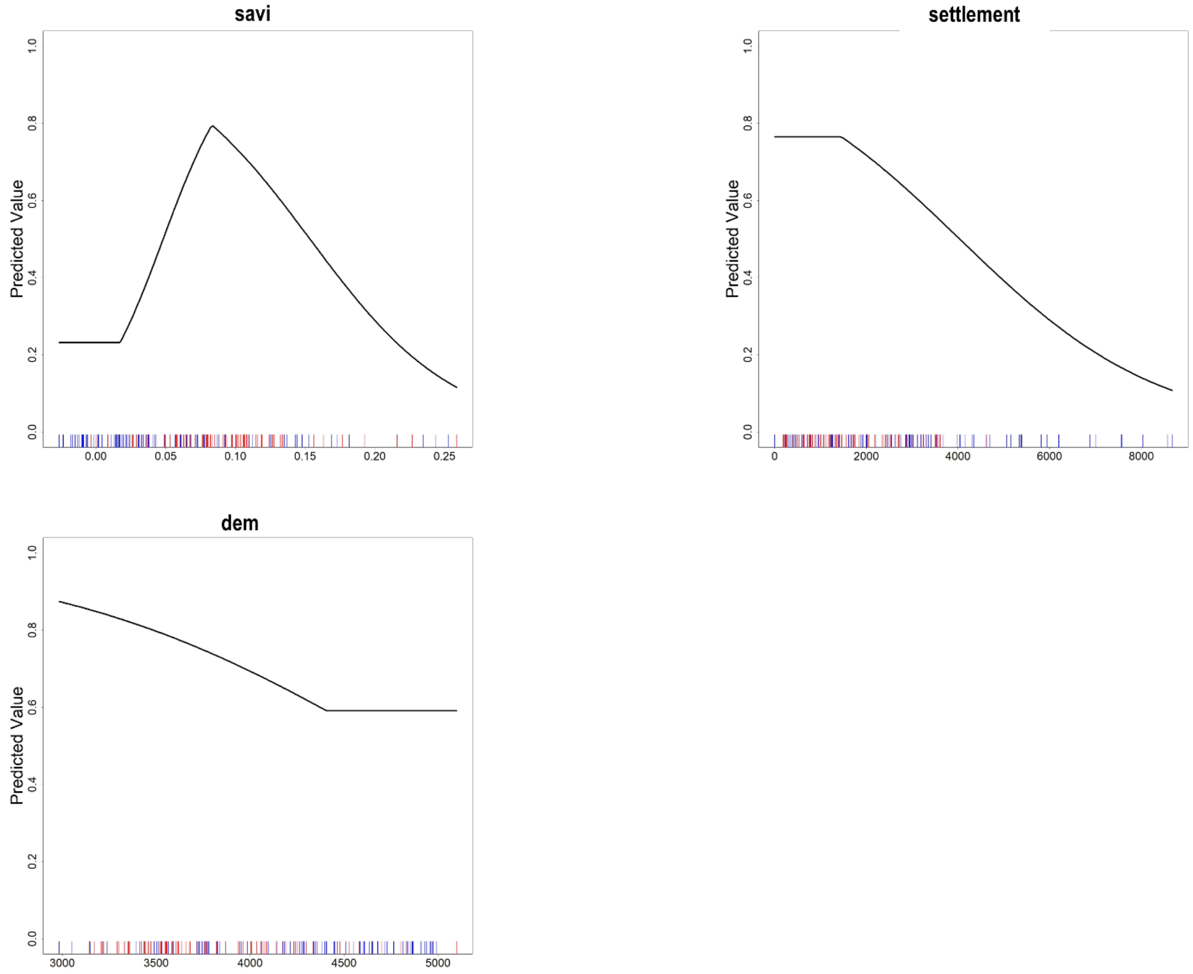

(I) Response curve of variables in MaxEnt model using Sentinel 2A derived LCLU classes and other variables.

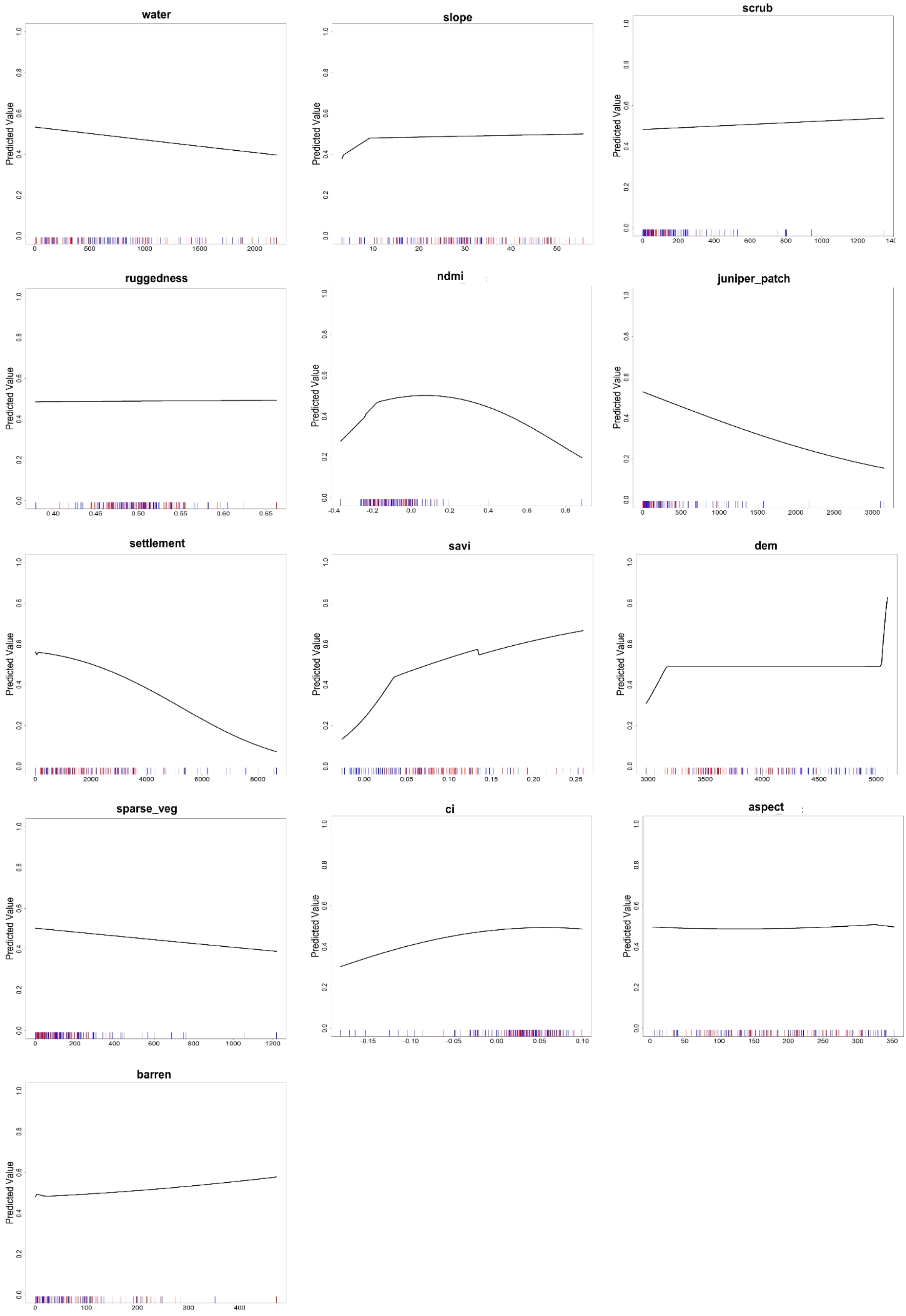

(J) Response curve of variables in RF model using Sentinel 2A derived LCLU classes and other variables.

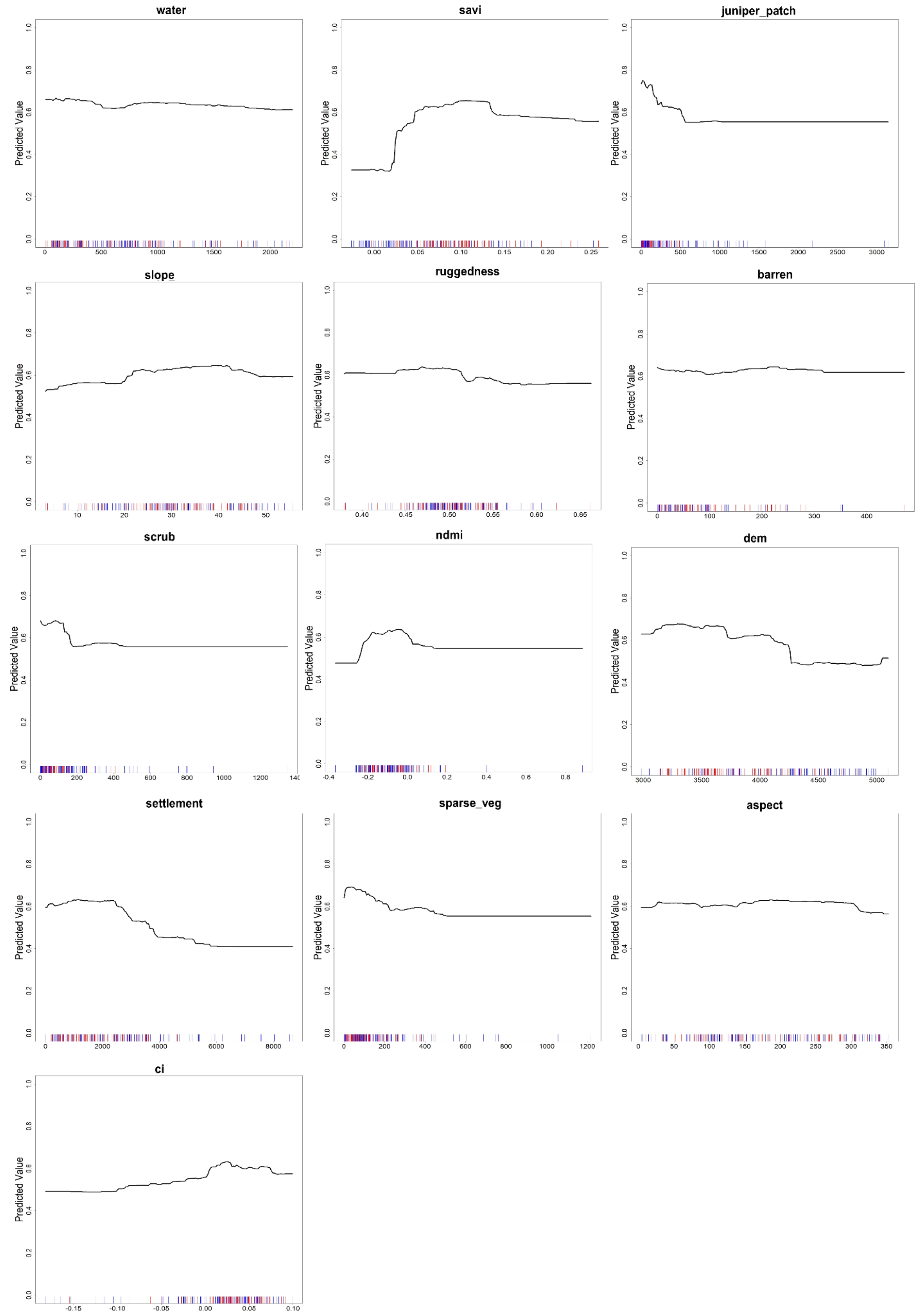

(K) Response curve of variables in BRT model using Integrated image derived LCLU classes and other variables.

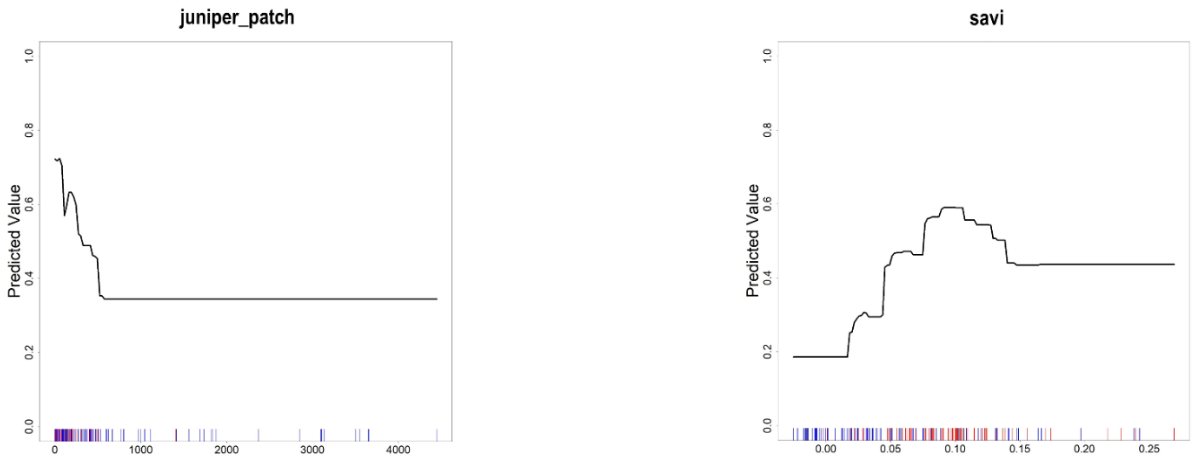

(L) Response curve of variables in GLM model using Integrated image derived LCLU classes and other variables.

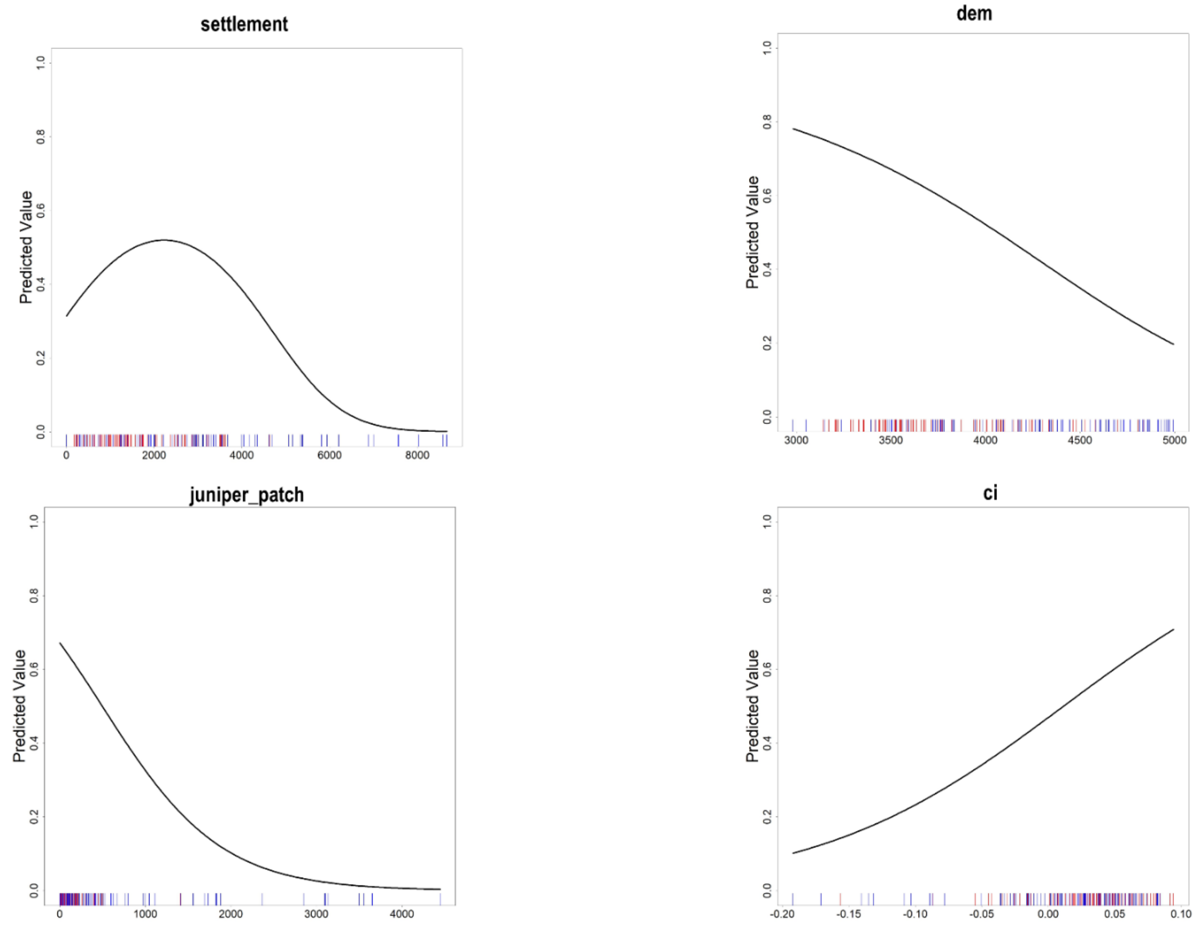

(M) Response curve of variables in MARS model using Integrated image derived LCLU classes and other variables.

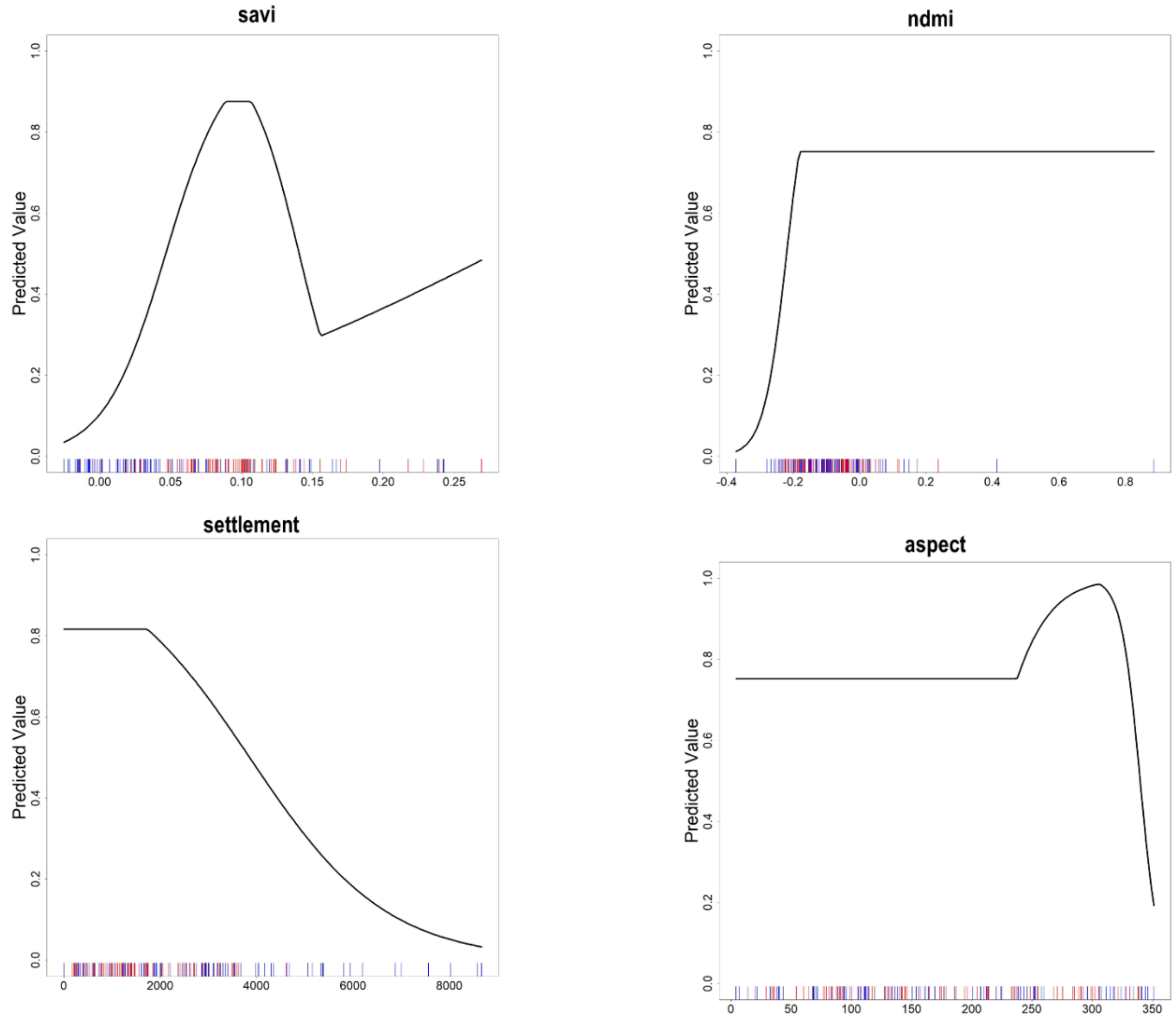

(N) Response curve of variables in MaxEnt model using Integrated image derived LCLU classes and other variables.

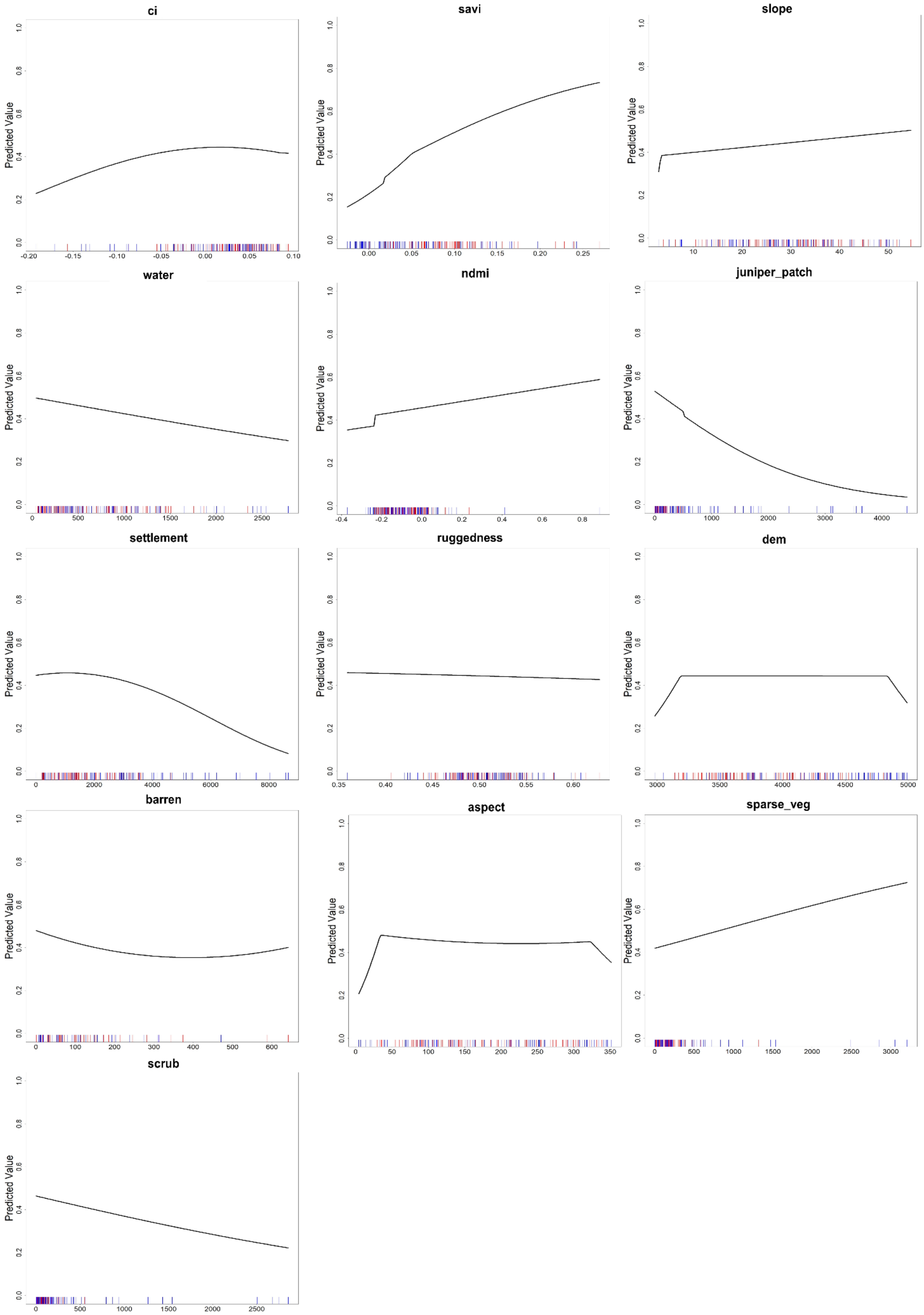

(O) Response curve of variables in RF model using Integrated image derived LCLU classes and other variables.

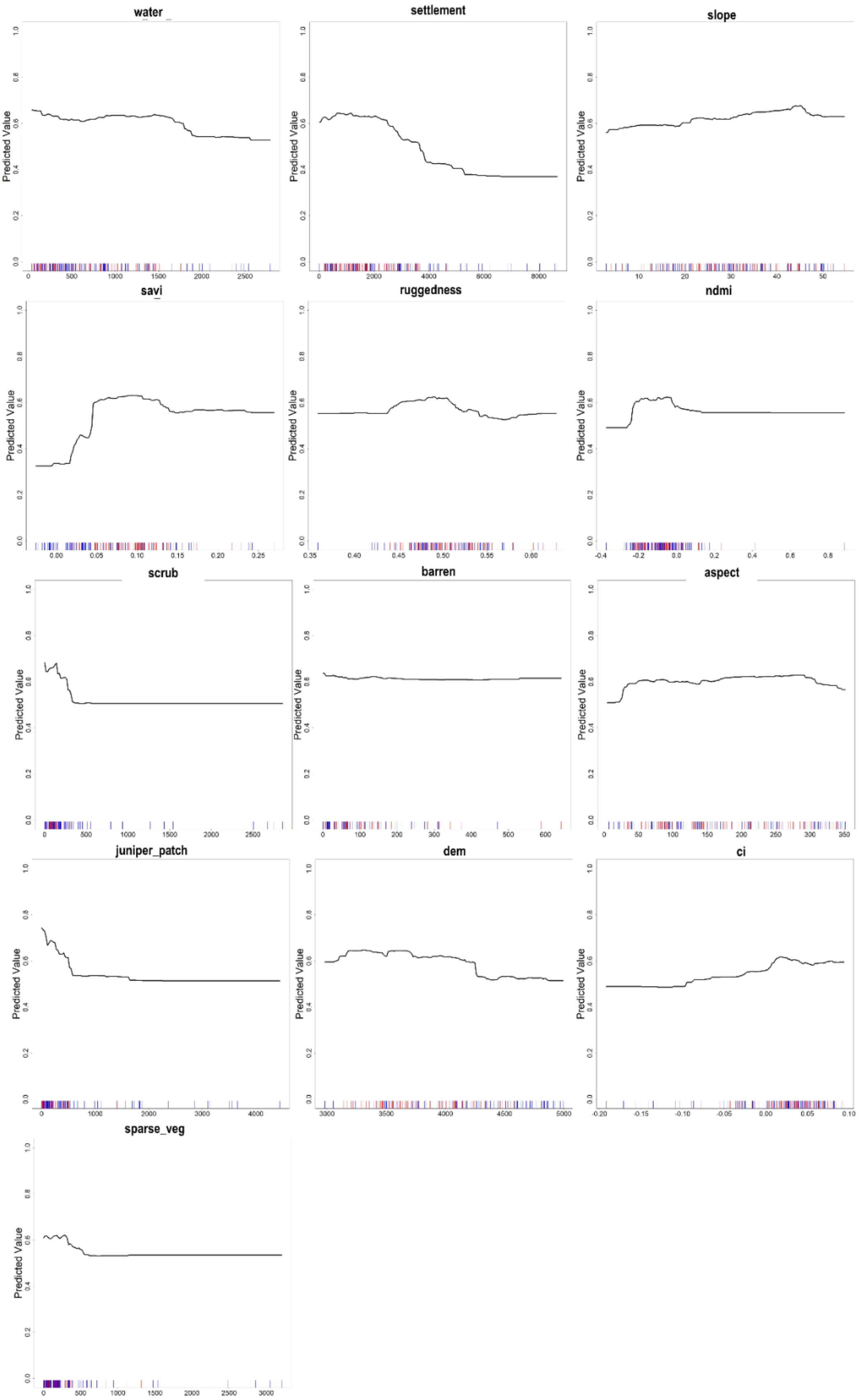

Supplement: S5 Fig — (PDF) [file pone.0306917.s007.pdf]
