# Supplementary figures and images for: Beyond traditional methods: Innovative integration of LISS IV and Sentinel 2A imagery for unparalleled insight into Himalayan ibex habitat suitability
Source: PLoS One. 2024 Oct 21;19(10):e0306917. doi: 10.1371/journal.pone.0306917 (PMC11493286; doi:10.1371/journal.pone.0306917)

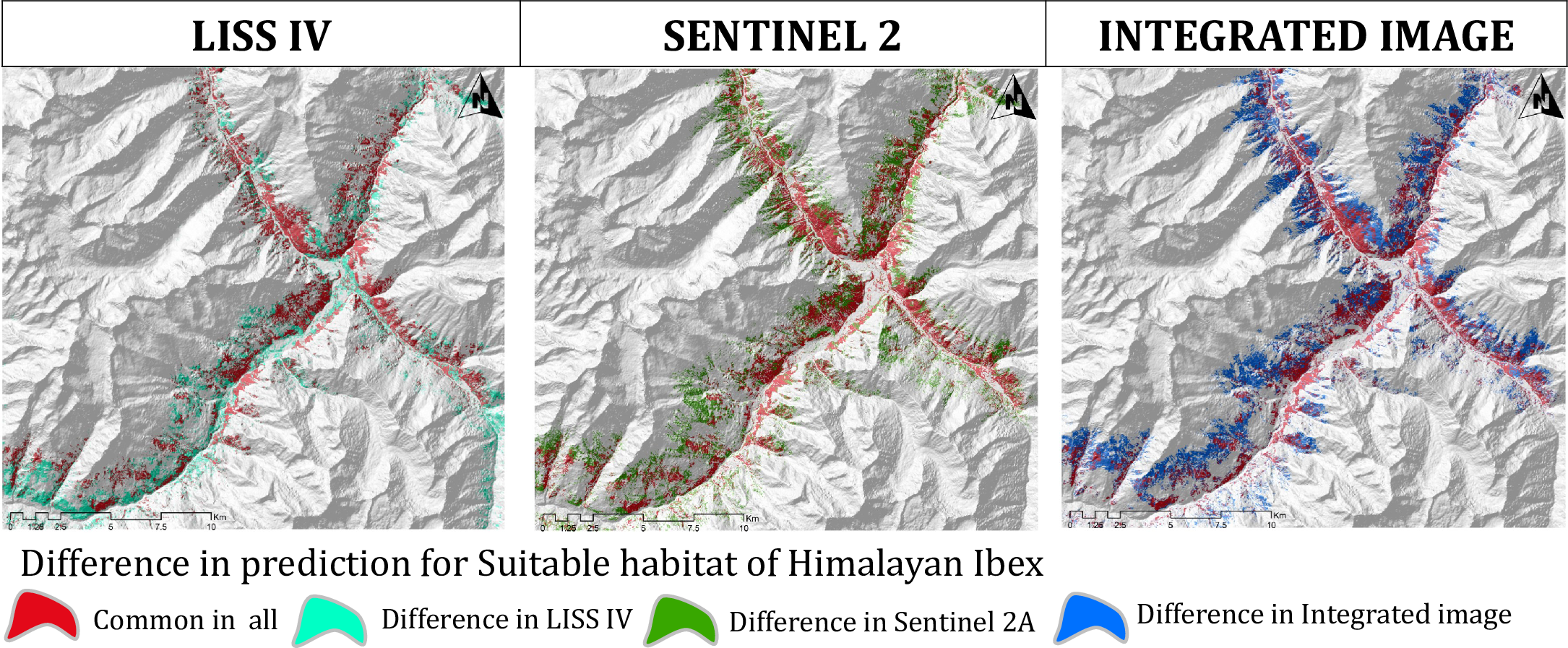

Supplement: S6 Fig — (TIF) [file pone.0306917.s008.tif]
